# Human striatal association megaclusters

**DOI:** 10.1152/jn.00387.2023

**Published:** 2024-03-20

**Authors:** Heather L. Kosakowski, Noam Saadon-Grosman, Jingnan Du, Mark C. Eldaief, Randy L. Buckner

**Affiliations:** ^1^Department of Psychology, Center for Brain Science, Harvard University, Cambridge, Massachusetts, United States; ^2^Department of Psychiatry, Massachusetts General Hospital, Charlestown, Massachusetts, United States; ^3^Athinoula A. Martinos Center for Biomedical Imaging, Massachusetts General Hospital, Charlestown, Massachusetts, United States; ^4^Department of Neurology, Massachusetts General Hospital, Charlestown, Massachusetts, United States

**Keywords:** association cortex, basal ganglia, fMRI, functional connectivity, striatum

## Abstract

The striatum receives projections from multiple regions of the cerebral cortex consistent with the role of the basal ganglia in diverse motor, affective, and cognitive functions. Within the striatum, the caudate receives projections from association cortex, including multiple distinct regions of prefrontal cortex. Building on recent insights about the details of how juxtaposed cortical networks are specialized for distinct aspects of higher-order cognition, we revisited caudate organization using within-individual precision neuroimaging initially in two intensively scanned individuals (each scanned 31 times). Results revealed that the caudate has side-by-side regions that are coupled to at least five distinct distributed association networks, paralleling the organization observed in the cerebral cortex. We refer to these spatial groupings of regions as striatal association megaclusters. Correlation maps from closely juxtaposed seed regions placed within the megaclusters recapitulated the five distinct cortical networks, including their multiple spatially distributed regions. Striatal association megaclusters were explored in 15 additional participants (each scanned at least 8 times), finding that their presence generalizes to new participants. Analysis of the laterality of the regions within the megaclusters further revealed that they possess asymmetries paralleling their cortical counterparts. For example, caudate regions linked to the language network were left lateralized. These results extend the general notion of parallel specialized basal ganglia circuits with the additional discovery that, even within the caudate, there is fine-grained separation of multiple distinct higher-order networks that reflects the organization and lateralization found in the cerebral cortex.

**NEW & NOTEWORTHY** An individualized precision neuroimaging approach reveals juxtaposed zones of the caudate that are coupled with five distinct networks in association cortex. The organization of these caudate zones recapitulates organization observed in the cerebral cortex and extends the notion of specialized basal ganglia circuits.

## Graphical abstract

**Figure:**
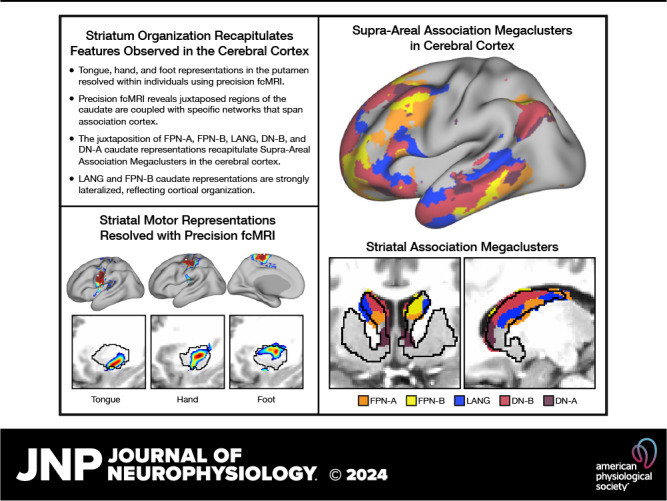

## INTRODUCTION

The basal ganglia support motor, affective, and cognitive functions through polysynaptic circuits that initiate in the cerebral cortex and return to cortex via the thalamus. Cortical projections to the striatum serve as the entry point for basal ganglia circuits (1). Providing a substrate for influences on broad functional domains, widely distributed association regions, including multiple regions within prefrontal cortex (PFC), project to extended zones of the striatum (2–4). In a landmark synthesis, Alexander, DeLong, and Strick (5) surmised that the basal ganglia support multiple distinct functions through specialized parallel closed-loop circuits (see also Ref. 81). They proposed five circuits that each maintain segregation within the basal ganglia and project back to distinct frontal territories, including circuits that could support cognitive functions.

Anterograde tracing studies in nonhuman primates specifically reveal that dorsolateral PFC (DLPFC) projects to a region in the head of the caudate extending through the internal capsule and into the ventral margin of the putamen (e.g., Ref. 3; see also Ref. 6 for additional convergent cases). One critical detail is that, depending on the specific region of PFC, the projection targets vary across the medial to lateral extent of the caudate, suggesting that there may be finer distinctions (3, 7, 8). However, fine-grained topographic differences between projections to the caudate have been difficult to resolve, and there are indications that distributed regions of PFC may converge in the striatum.

Dual tracer injections from frontal and parietal regions yield substantially overlapping (interdigitated) striatal projection patterns in some cases and in other cases minimal overlap (3). In a thorough quantitative analysis of 34 tracer injections in macaque PFC, Averbeck et al. (9) noted variation in the patterning of projections to the striatum as well as an overlapping zone in the medial caudate that receives projections from multiple distinct regions of PFC (see also Ref. 6). Exploring whether multiple, independent networks associated with higher-order cognition have distinct representations within the caudate is the focus of the present study. The caudate is small, and the topography of the cortical inputs is incompletely understood, yet the distinctions among cortical association networks that have recently emerged provide an impetus to revisit striatal organization with precision within-individual approaches.

Providing a foundation for our work, human neuroimaging studies using functional connectivity MRI (fcMRI) reliably find that PFC networks include the caudate (10–17). For example, Choi et al. (11) analyzed data from 1,000 individuals and found that the caudate was robustly correlated with networks that involve distributed PFC regions. However, fine distinctions between association networks were not apparent. Seed regions placed in a PFC network linked to putative cognitive control involving DLPFC and a region along the frontal midline involved in distinct functions displayed substantially overlapping patterns of correlation in the caudate (see also Ref. 6). Such an observation is consistent with the possibility of convergence but also may simply be due to blurring that emerges when anatomical differences between individuals are averaged (see Ref. 18 for discussion).

Gordon et al. (13) recently explored striatal organization within intensively sampled individual participants (see also Ref. 18). They observed patterns generally consistent with the prior group-averaged analyses but also resolved spatial details that were not evident in prior work. Critically, regions associated with anterior, dorsal, and ventral zones of the caudate showed partially nonoverlapping patterns in PFC. Contrasting an anterior region of the striatum with a dorsal region in an exemplar dataset specifically separated networks putatively involved in language functions from others. Greene et al. (18) noted separation in the caudate between regions linked to a PFC network implicated in cognitive control and a distinct zone associated with the network commonly known as the default network. In quantitative analyses of overlap and separation among individuals, Greene et al. (18) further concluded that the caudate possesses multiple network-specific zones. These findings represent a level of anatomical specificity not possible when averaging group data, which we build upon here.

The present work utilizes a precision within-individual approach to explore in detail the striatal topography linked to distributed higher-order association networks. We utilize three specific advances to pursue this work. First, we analyze individual participants who were scanned extensively across many sessions to boost the signal-to-noise ratio (SNR). The data come from Du et al. (19) and are reanalyzed here with focus on the striatum. All of the analyses are conducted using the idiosyncratic anatomy of the individual. Second, we register the images across sessions, using a processing pipeline optimized for within-individual registration and minimization of ancillary spatial blurring (20). Third, we base our explorations on the observation of spatially precise side-by-side juxtapositions of association networks across the cerebral cortex, specifically focusing on five networks that are highly replicable and functionally dissociable (19, 20). These five juxtaposed networks form supra-areal association megaclusters (SAAMs) within multiple zones of the cerebral cortex. Here we leverage these recent insights from precision explorations of the cerebral cortex to discover novel representations of multiple association networks within the human caudate.

## METHODS

### Participants

Data from participants labeled *S1* and *S2* were utilized (*N* = 2; age = 22 and 23 yr, both women). These data have been previously reported in Xue et al. (21) with a focus on the cerebellum and in Du et al. (19) with a focus on the cerebral cortex. Here, the data were reanalyzed with a focus on the striatum. Data from additional participants labeled *P1–P15* from Du et al. (19) were also utilized to generalize the findings discovered in the initial two participants (*N* = 15; mean age = 22.1 yr, SD = 3.9 yr, 9 women). Participants were right-handed, native English speakers with no history of neurological and psychiatric illness. Participants provided informed consent in-line with protocols approved by the Institutional Review Board of Harvard University.

### MRI Data Acquisition

Data were acquired at the Harvard Center for Brain Science with a 3-T Siemens MAGNETOM Prisma^fit^ MRI scanner. Acquisition methods have been described previously (19), and relevant portions are repeated here. Data were collected with the vendor-supplied 64-channel and 32-channel phased-array head-neck coils (Siemens Healthineers, Erlangen, Germany). Data from the two coils are nearly indistinguishable for functional acquisitions and have been combined previously (19, 22), and they are also combined here. Head motion was mitigated with foam and inflated padding. Participants were instructed to remain still and awake and to look at a rear-projected display through a custom-built mirror attached to the head coil. During functional scanning, participants fixated a centrally presented plus sign (black on a gray background). The scanner room was illuminated, and eyes were video recorded with an EyeLink 1000 Plus with Long-Range Mount (SR Research, Ottawa, ON, Canada). Alertness was scored during each functional run.

*S1* and *S2* each participated in 31 MRI sessions with 61 or 62 resting-state fixation runs in total acquired per individual. Each session involved multiple resting-state fixation runs acquired with blood oxygenation level-dependent (BOLD) contrast (23, 24). A custom multiband gradient-echo echo-planar pulse sequence developed by the Center for Magnetic Resonance Research (CMRR) at the University of Minnesota (Refs. 25–27; see also Ref. 28) was used: voxel size = 2.4 mm, repetition time (TR) = 1,000 ms, echo time (TE) = 32.6 ms, flip angle = 64°, matrix 88 × 88 × 65, anterior-to-posterior (AP) phase encoding, multislice 5× acceleration. Sixty-five slices were automatically positioned (29). Signal dropout was minimized by selecting a slice 25° from the anterior-posterior commissural plane toward the coronal plane (30, 31). Each run lasted 7 min 2 s (422 frames, with 12 frames removed for T1 equilibration). No generalized autocalibrating partial parallel acquisition (GRAPPA) acceleration was used, which, when combined with multiband acceleration, can cause poor data quality (32). A dual-gradient-echo B0 field map was acquired to correct for spatial distortions: TE = 4.45 and 6.91 ms; slice prescription/resolution matched to the BOLD sequence.

*P1–P15* each participated in 8–11 sessions with 15–24 resting-state fixation runs in total acquired per individual. BOLD acquisition parameters were similar to those used for *S1* and *S2* except the matrix was 92 × 92 × 65 [field of view (FOV) = 221 × 221]. For *P12*, the first two sessions were acquired with a different FOV (211 × 211); as such, the matrix was 88 × 88 × 65, matching *S1* and *S2*. Each fixation run was 7 min 2 s (422 frames, first 12 removed for T1 equilibration).

For *S1* and *S2*, at least one rapid T1-weighted (T1w) structural scan was collected for each participant with a multiecho magnetization prepared rapid acquisition gradient echo (ME-MPRAGE) three-dimensional (3-D) sequence (29): voxel size = 1.2 mm, TR = 2,200 ms, TE = 1.57, 3.39, 5.21, 7.03 ms, TI = 1,100 ms, flip angle = 7°, matrix 192 × 192 × 176, in-plane GRAPPA acceleration = 4. For P1–P15, high-resolution T1w and matched T2-weighted (T2w) scans were also acquired using Human Connectome Project (HCP) parameters. The T1w HCP ME-MPRAGE structural scan used voxel size = 0.8 mm, TR = 2,500 ms, TE = 1.81, 3.60, 5.39, and 7.18 ms, TI = 1,000 ms, flip angle = 8°, matrix 320 × 320 × 208, 144, in-plane GRAPPA acceleration = 2. The T2w HCP structural scan used a sampling perfection with application-optimized contrasts using different flip angle evolution sequence (SPACE; Siemens Healthineers, Erlangen, Germany): voxel size = 0.8 mm, TR = 3,200 ms, TE = 564 ms, 208 slices, matrix = 320 × 300 × 208, in-plane GRAPPA acceleration = 2.

Before inclusion in any analysis, all resting-state fixation runs were screened for quality as described in Du et al. (19). All data exclusions were finalized before analyses of functional connectivity responses to ensure that data exclusion was unbiased by resulting data patterns.

### Data Processing and Registration That Minimizes Spatial Blurring

Data were processed with an in-house preprocessing pipeline (“iProc”) that preserved spatial details by minimizing spatial blurring and the number of interpolations [described in detail in Braga et al. (20)]. For *S1* and *S2*, the preprocessed data were taken from Xue et al. (21) and additionally processed with the 15-network cerebral network estimates reported in Du et al. (19). For *P1–P15*, the preprocessed data and network estimates for the cerebral cortex were taken directly from Du et al. (19). Descriptions of relevant processing methods are repeated here.

Data were interpolated to a 1-mm isotropic native-space atlas (with all processing steps composed into a single interpolation) that was then projected with FreeSurfer v6.0.0 to the fsaverage6 cortical surface (40,962 vertices per hemisphere; Refs. 33, 34). Five transformation matrices were calculated: *1*) a motion correction matrix for each volume to the run’s middle volume [linear registration, 6 degrees of freedom (DOF); MCFLIRT, FSL], *2*) a matrix for field map-unwarping the run’s middle volume, correcting for field inhomogeneities caused by susceptibility gradients (FUGUE, FSL), *3*) a matrix for registering the field map-unwarped middle BOLD volume to the within-individual mean BOLD template (12 DOF; FLIRT, FSL), *4*) a matrix for registering the mean BOLD template to the participant’s T1w native-space image (6 DOF; using boundary-based registration, FreeSurfer), and *5*) a nonlinear transformation to MNI space (nonlinear registration; FNIRT, FSL). The individual-specific mean BOLD template was created by averaging all field map-unwarped middle volumes after being registered to an upsampled 1.2 mm and unwarped midvolume template (interim target, selected from a low-motion run acquired close to a field map). The T1w native-space template was then resampled to 1.0-mm isotropic resolution.

Confounding variables including six head motion parameters, whole brain, ventricular signal, deep cerebral white matter signal, and their temporal derivatives were calculated from the BOLD data in T1w native space. The signals were regressed out from the BOLD data with 3dTproject, AFNI (35, 36). The residual BOLD data were then band-pass filtered at 0.01–0.10 Hz with 3dBandpass, AFNI (35, 36). For surface analyses, the native-space data were resampled to the fsaverage6 standardized cortical surface mesh with trilinear interpolation and then surface-smoothed with a 2-mm full-width-at-half-maximum (FWHM) Gaussian kernel. For striatal analyses, MNI volume space data were smoothed with a 4-mm FWHM Gaussian kernel. The iProc pipeline thus allowed for robustly aligned BOLD data, with interpolation minimized. Relevant final output spaces included the MNI volume space and the fsaverage6 cortical surface.

### Temporal Signal-to-Noise Ratio Maps

Data using BOLD-contrast (T2* images) and echo-planar imaging result in variable distortion and signal dropout due to magnetic susceptibility artifacts (37). This may be a particular challenge for studies of the striatum given the proximity of the ventral striatum to the regions of susceptibility artifact near the sinuses. It is thus important to examine the temporal signal-to-noise (tSNR) maps in the volume to ensure that adequate signal properties are present in the regions of the striatum being examined. Voxelwise tSNR maps were computed for each participant. tSNR was calculated for each voxel in MNI atlas space by dividing the mean time course within a functional MRI (fMRI) run by its standard deviation before whole brain signal regression and then averaged across runs. tSNR maps were examined in and around the striatum.

### Striatum Identification and Visualization

A key step for the present inquiry was to ensure high-quality parcellations of the striatum within individual participants. To isolate striatal voxels, we identified the caudate, putamen, and nucleus accumbens (NAc) in each participant with the FreeSurfer automated parcellation (33). The FreeSurfer-based masks were combined, binarized, and warped to MNI space with the same individual-specific matrix used in iProc preprocessing. Striatum masks were created for different purposes, which included *1*) visualization of the striatal boundaries, *2*) parcellation of the striatum allowing for voxel assignments that edge just past the striatal T1w boundaries,^1^ and *3*) visualization of the cortex adjacent to the striatum. To accommodate the different uses of striatal masks, the boundary of the striatum was dilated at different levels with *fslmaths -dilM* and binarized. 1× Dilation was used for striatal boundary visualization, 3× dilation was used for striatal parcellation, and 5× dilation was used to visualize the adjacent cerebral cortex. The 5× dilation mask was specifically used in control checks to determine the effect of signal blurring of the cerebral cortex into the striatum.

### Within-Individual Striatum-to-Cortex Correlation Matrices

For each participant, the pairwise Pearson correlation coefficients between the fMRI time courses at each surface vertex were calculated for each fixation fMRI run, yielding an 81,924 × 81,924 matrix (40,962 vertices/hemisphere). Separately, the pairwise Pearson correlation coefficients between the fMRI time courses at each voxel within a 3× dilated striatum mask and each cortical vertex were calculated for each fixation fMRI run, yielding a 68,713 × 81,924 matrix. The matrix was then Fisher *r*-to-*z* transformed and averaged across all runs to yield a single best estimate of the within-individual correlation matrix. This *z*-scored matrix was used to explore network organization. The mean correlation maps were assigned to a cortical and subcortical template combining left and right hemispheres of the fsaverage6 surface and subcortex around the striatum of the MNI152 volume into the CIFTI format to interactively explore correlation maps with the Connectome Workbench’s wb_view software (38, 39). The color bar scales of correlation maps were thresholded to highlight individual-specific anatomy with the Jet lookup table for visualization. Correlation maps are additionally provided on BALSA (https://balsa.wustl.edu/) to enable viewing in alternative color scales.

### Validation of the Approach for Striatal Mapping Using Somatomotor Topography

The goal of our study was to discover novel representations of association networks within the striatum. To validate that our data and processing methods can estimate cortical-striatal organization, we began by first exploring correlations between regions in the primary motor cortex and the putamen. This exploration was conducted as a positive control [extending from Choi et al. (11)]. Separate representations of body effectors have been well established via direct anatomical explorations in nonhuman primates (40, 41). For this control analysis, seed regions were identified in the approximate locations of the foot, hand, and tongue motor representations of the cerebral cortex. Then the correlations were visualized in the striatum.

### Individualized Network Estimates of the Cerebral Cortex

Striatal mapping began by utilizing within-individual precision maps of network organization in the cerebral cortex. For all participants, the 15-network cerebral cortex estimates were taken directly from Du et al. (19), with relevant method descriptions repeated here. A multisession hierarchical Bayesian model (MS-HBM) was implemented to estimate the cortical networks (42). For *S1* and *S2*, the resting-state fixation data were split into three subsets. For *S1*, *dataset 1* included *runs 1–20*, *dataset 2* included *runs 21–40*, and *dataset 3* included *runs 41–62*. For *S2*, *dataset 1* included *runs 1–20*, *dataset 2* included *runs 21–40*, and *dataset 3* included *runs 41–61*. The MS-HBM was independently implemented on the first two subsets (*datasets 1* and *2*). *Dataset 3* was set aside to allow for seed region-based fcMRI analysis to confirm network specificity. For *P1–P15*, the MS-HBM was implemented on each participant’s full set of resting-state fixation runs.

To estimate networks in the cerebral cortex, the connectivity profile of each vertex on the cortical surface was first estimated as its functional connectivity to 1,175 regions of interest (ROIs) that uniformly distributed across the fsaverage6 surface meshes (43). For each run of data, the Pearson’s correlation coefficients between the fMRI time series at each vertex (40,962 vertices/hemisphere) and the 1,175 ROIs were computed. The resulting 40,962 × 1,175 correlation matrix per hemisphere was then binarized by keeping the top 10% of the correlations to obtain the functional connectivity profiles.

Next, the MS-HBM was initialized with a group-level parcellation estimated from a subset of the HCP S900 data release using the clustering algorithm from Yeo et al. (43). The group-level parcellation was used to initialize the expectation-maximization algorithm for estimating parameters in the MS-HBM. The goal of applying the model as used here was to obtain the best estimate of networks within each individual participant’s dataset, not to train parameters and apply them to unseen data from new participants (e.g., Ref. 42).

The 15-network estimates include Somatomotor-A (SMOT-A), Somatomotor-B (SMOT-B), Premotor-Posterior Parietal Rostral (PM-PPr), Cingulo-Opercular (CG-OP), Salience/Parietal Memory Network (SAL/PMN), Dorsal Attention Network-A (dATN-A), Dorsal Attention Network-B (dATN-B), Frontoparietal Network-A (FPN-A), Frontoparietal Network-B (FPN-B), Default Network-A (DN-A), Default Network-B (DN-B), Language (LANG), Visual Central (VIS-C), Visual Peripheral (VIS-P), and Auditory (AUD).

Here we focus on five higher-order association networks that are juxtaposed throughout the cerebral cortex: FPN-A, FPN-B, LANG, DN-B, and DN-A.

### Individual-Specific Parcellation of the Striatum

To parcellate the striatum, we adapted a winner-takes-all strategy (11, 21). The objective of the parcellation was to assign each striatal voxel to its most strongly correlated cortical network. The correlation strength between each voxel in the striatum and each vertex on the cortical surface was computed. Then, for each voxel in the striatum, we identified the 400 surface vertices that had the strongest correlation to that voxel. We estimated the percentage of MS-HBM network assignments of the 400 surface vertices and assigned the voxel to the network with the highest proportion of network assignments.

For *S1* and *S2* the separate datasets within each individual allowed for discovery, replication, and validation analyses to be performed. Specifically, *datasets 1* and *2* were used as discovery and replication datasets for the winner-takes-all striatum parcellation, and *dataset 3* was set aside and used for the model-free seed region-based fcMRI analysis (see below).

Although our focus was on five association networks (FPN-A, FPN-B, LANG, DN-B, and DN-A), it is important to note that the striatal voxels were not constrained to be assigned solely to these networks. Rather, voxels were permitted to be assigned to their most correlated network among all 15 of the cortical networks in either hemisphere. That is, every voxel in the striatum had an opportunity to be assigned to any of the 15 estimated cortical networks.

### Model-Free Seed Region-Based Exploration of Striatal Network Assignments

A winner-takes-all approach makes a strong assumption that striatal voxels are associated with a single cortical network and thus could bias estimates to detect segregation rather than convergence. For example, a striatal voxel correlated at a roughly similar level to three separate cortical networks will obligatorily be assigned to a single network, hiding the critical detail that the voxel is correlated with a broad extent of the cerebral cortex (see also Ref. 18). To provide another means to assess the specificity of networks linked to the striatum, the set-aside *dataset 3* from *S1* and *S2* was used prospectively to test for network correlation patterns. This analysis provided for an independent confirmation of the network assignments using a distinct method in separate data.

Specifically, the parcellations for *S1* and *S2* from *datasets 1* and *2* were used to guide seed region placement. In the independent, left-out *dataset 3* from the same participant, seed regions were placed in zones of the caudate that belonged to FPN-A, FPN-B, LANG, DN-B, and DN-A. We then tested whether the seed regions would recapitulate the distributed correlation pattern within each network’s boundaries in the cerebral cortex.

As a complementary analysis, we also explored the raw correlation patterns in the striatum from multiple seed regions placed in the cerebral cortex. Specifically, seed regions were placed in the MS-HBM-defined cortical networks using the composite set of all fMRI data. For each network, we placed one seed region in four different spatially distributed nodes of the cortical network and observed correlation patterns in the striatum that corresponded to voxel assignments from the winner-takes-all parcellation strategy. The expectation is that raw correlation patterns within the striatum will be spatially diffuse given limitations of resolution but should also reveal a spatial organization that is consistent with the parcellation. Moreover, distinct seed regions in the cortex from the same network should reveal convergent striatal patterns, and these convergent patterns should be distinct from the sets of seed regions positioned within distinct networks. That is, the raw correlation patterns of seed regions in the cortex should reveal patterning in the striatum consistent with the automated network parcellation.

### Adjustment for Signal Blurring from Adjacent Cortex

The striatum is close to the cortical surface, particularly along the frontal midline and where the cortex of the insula folds into the Sylvian fissure. This proximity can cause erroneous assignments within the striatum that are due to signal blur from the surrounding cortical surface (see Refs. 11, 14 for similar considerations). To understand the potential impact of signal blur, we conducted a series of control analyses.

In these control analyses, we dilated the striatal mask from *S1* and *S2* such that it extended beyond the anatomical boundary of the striatum into the adjacent cortical regions and ventricles. For this visualization, we removed correlation values below a specified threshold. Specifically, for the top 400 voxel-to-vertex correlations, if a correlation was below a predetermined threshold [e.g., *r*(*z*) < 0.10], the vertex was excluded from the count. For example, if 100 of the 400 vertices had a correlation value below the threshold, only the 300 vertices with correlations above the threshold would be included to determine the assignment. If all 400 voxel-to-vertex correlation values were below the threshold, the voxel did not receive a network assignment. Using a high correlation value as our threshold and a mask that extended into the adjacent cortical ribbon enabled us to visualize cortical signal bleed into the striatum. We tested thresholds *r* = 0.01 and *r* = 0.05–0.40 in increments of 0.05 across unsmoothed and smoothed data to visualize whether any of our interpreted observations were potentially impacted by spatial blur.

### Prospective Replication

As the results reveal, the caudate was discovered to contain a megacluster of five closely juxtaposed regions, each coupled to a distinct cerebral network. Given the novelty of this observation and that it was found in only two initial individuals, we sought generalization to additional individuals. Data from *P1–P15* were used to replicate the findings. In each of the 15 individuals, the same procedures were employed as for *S1* and *S2* to parcellate the striatum, using one high-quality dataset available per individual. Note again that any voxel within the striatum could be assigned to any of the 15 networks. Thus, assignment to the five association networks (FPN-A, FPN-B, LANG, DN-B, and DN-A) in a clustered pattern within the caudate was not obligated.

### Laterality Analysis

Several association networks are lateralized in the cerebral cortex, meaning that on average they tend to have a greater representation in one hemisphere than the other. The LANG network is a well-documented example with a spatially larger representation in the left hemisphere of the cerebral cortex. An open question is whether the laterality of networks in the stratum parallels the laterality observed in the cerebral cortex.

As a post hoc exploration, the number of vertices/voxels assigned to each network in each hemisphere was computed for the cerebral cortex and the striatum. This was done separately for *S1* and *S2* and for *P1–P15*, providing two estimates of network lateralization. For striatal counts, we used a caudate mask to ensure that we isolated voxels with clear striatal signal. Then, to account for the fact that there was an uneven number of voxels in each hemisphere of the striatum, the proportion of vertices/voxels was computed by dividing the raw count of voxels/vertices assigned to each network by the total number of vertices/voxels in each hemisphere.

### Software and Statistical Analysis

Functional connectivity was calculated in MATLAB (version 2019a; MathWorks, Natick, MA) using Pearson’s product moment correlations. FreeSurfer v6.0.0, FSL, and AFNI were used during data processing. The estimates of networks on the cortical surface were visualized in Connectome Workbench v1.3.2. Model-free seed region confirmations were performed in Connectome Workbench v1.3.2. Network parcellation was performed using code from Ref. 42 on GitHub (https://github.com/ThomasYeoLab/CBIG/tree/master/stable_projects/brain_parcellation/Kong2019_MSHBM). All data are publicly available on NIMH Data Archive (NDA); striatal parcellations are available on BALSA (https://balsa.wustl.edu/); data for Fig. 8 and all code are available on Open Science Framework (OSF; https://osf.io/rhbfn/).

## RESULTS

### Most of the Striatum Possesses Good Signal-to-Noise Properties

**Figure 1.**
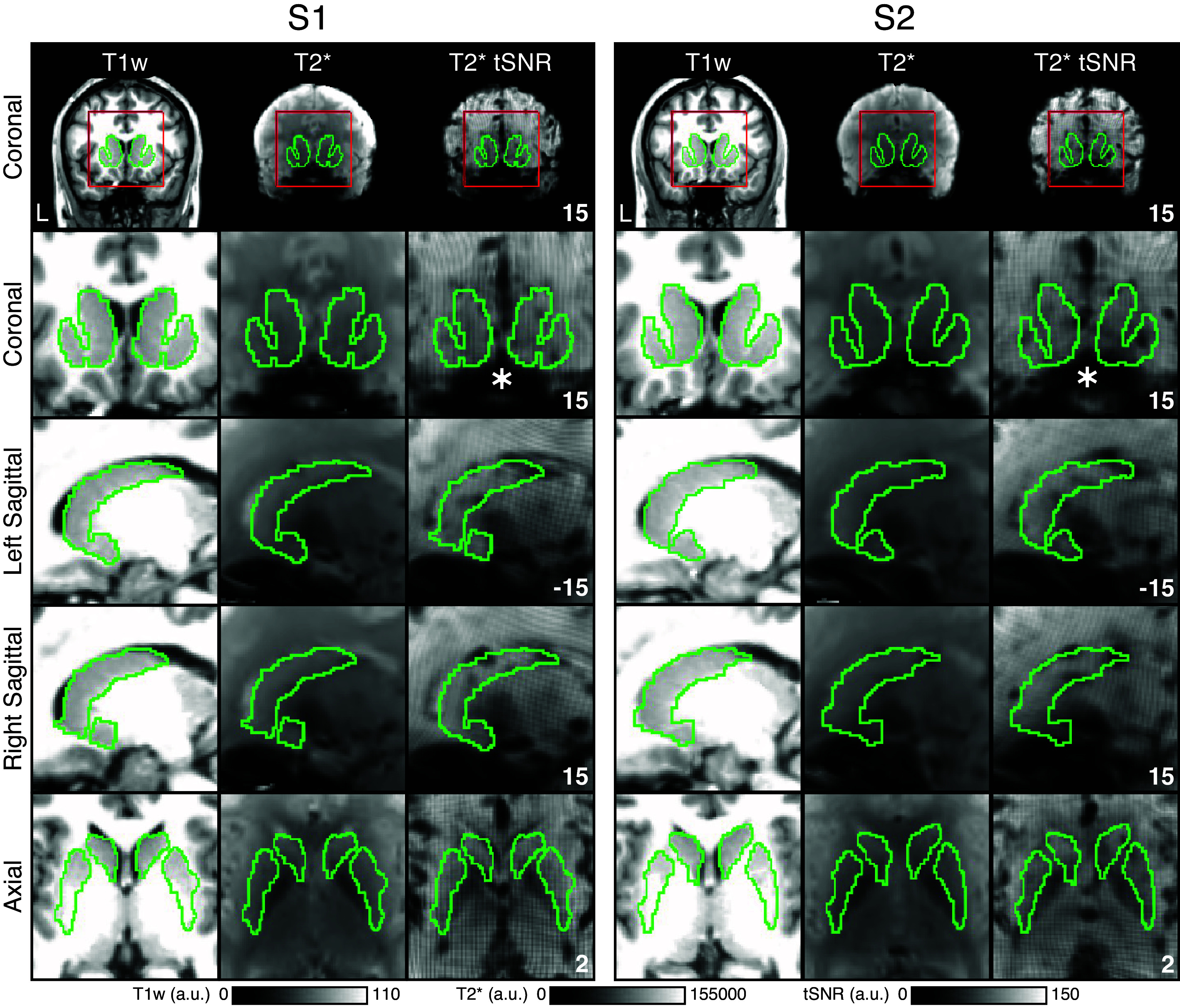
The striatum possesses good signal-to-noise properties. Within-individual striatal boundaries in MNI atlas space are shown for *S1* and *S2*. *Top*: whole brain coronal slices. The red line indicates the bounding box used for striatal visualizations. The green line depicts the estimated boundaries of the striatum identified within each individual’s T1-weighted (T1w) structural image and then transformed to MNI atlas space. *Bottom*: estimated striatal boundaries are overlaid on T1w (*left*), mean T2* (*center*), and mean temporal signal-to-noise ratio (tSNR, *right*) images to illustrate distortion and signal properties. There is good tSNR throughout much of the striatum including the caudate. There is notable signal drop in the ventral striatum near the region of the susceptibility artifact (indicated with asterisks). Coordinates at *bottom right* of panels indicate the slice in the MNI152 atlas. a.u., Arbitrary units.

For each participant, the caudate, putamen, and NAc were isolated. Figure 1 illustrates the striatal boundaries in relation to the T1w structural data, the mean BOLD (T2*), and the mean tSNR maps for *S1* and *S2* (see Supplemental Figs. S1–S8 for *P1–P15*). Because of the dense within-individual fMRI data sampling, there was sufficient signal in most regions of the striatum for thorough exploration, including good tSNR in the caudate. Signal dropout was present near to and within the ventral striatum/NAc. The affected regions of the striatum were not the focus of the present study.

### Somatomotor Topography Is Observed in the Striatum

**Figure 2.**
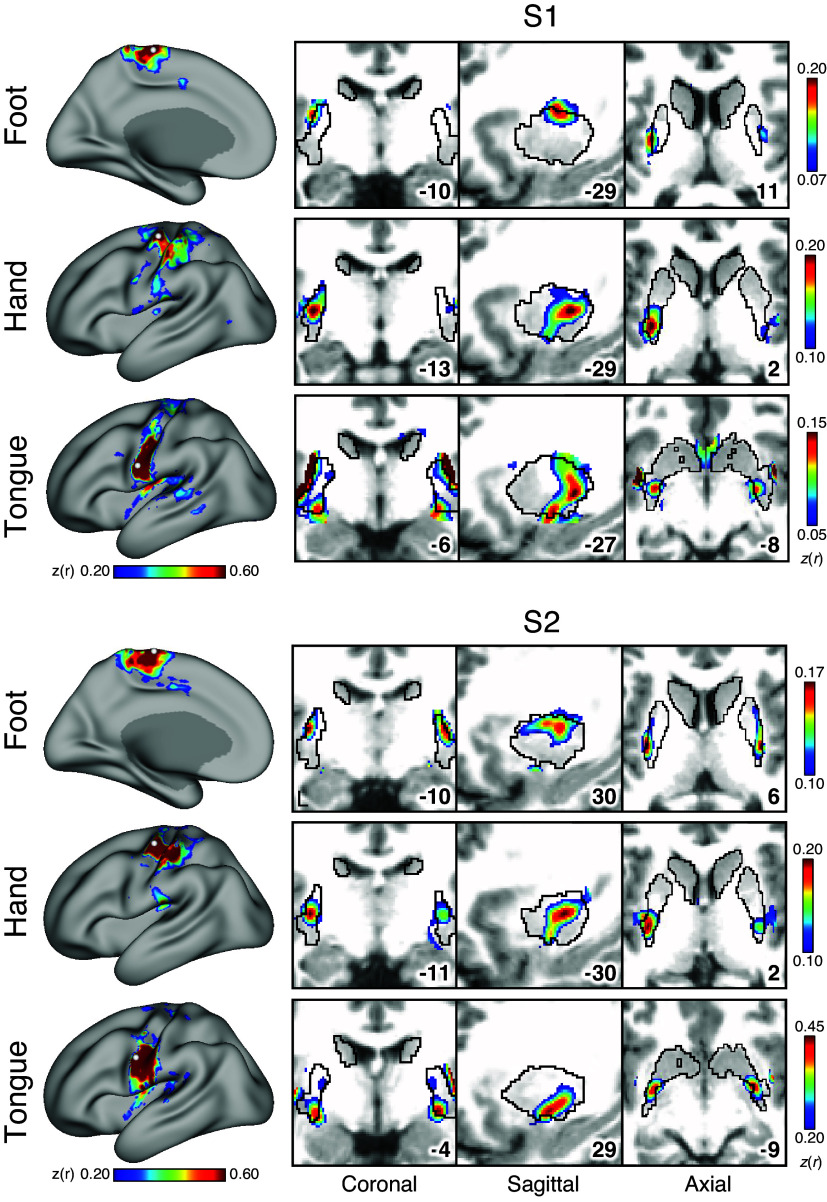
Somatomotor organization is observed in the striatum. Seed regions were placed in the approximate location of the foot, hand, and tongue motor representations of the cerebral cortex, and correlations were visualized in the striatum (*S1*, *top*; *S2*, *bottom*). The observed pattern is consistent with well-established striatal somatomotor topography. All correlation values are visualized with the jet color scale; surface thresholds are *z*(*r*) = 0.20–0.60; striatal thresholds are individually adjusted and indicated on the *right*. Coordinates at *bottom right* of panels indicate the slice in the MNI152 atlas.

We constructed striatal somatomotor maps to validate that our fcMRI methods possess sensitivity and specificity to resolve fine-grained topography within individual participants. Cortical seed regions were placed in the approximate locations of the foot, hand, and tongue motor representations (Fig. 2, *left*). Then, the correlations in the striatum were visualized. In both *S1* and S2, foot, hand, and tongue region correlations were present in the putamen with the expected spatial organization (Fig. 2, *right*). Specifically, the cortical foot region was correlated with a dorsolateral portion of the putamen; the cortical tongue region was correlated with a ventromedial portion of the putamen; and the cortical hand region was correlated with a portion of the putamen that was between the foot and tongue representations. This result, a form of positive control, demonstrates that there is sufficient signal and spatial specificity to observe well-established striatal subregions.

### Striatal Parcellations Reveal Close Juxtaposition of Subregions Linked to Distinct Cortical Association Networks

**Figure 3.**
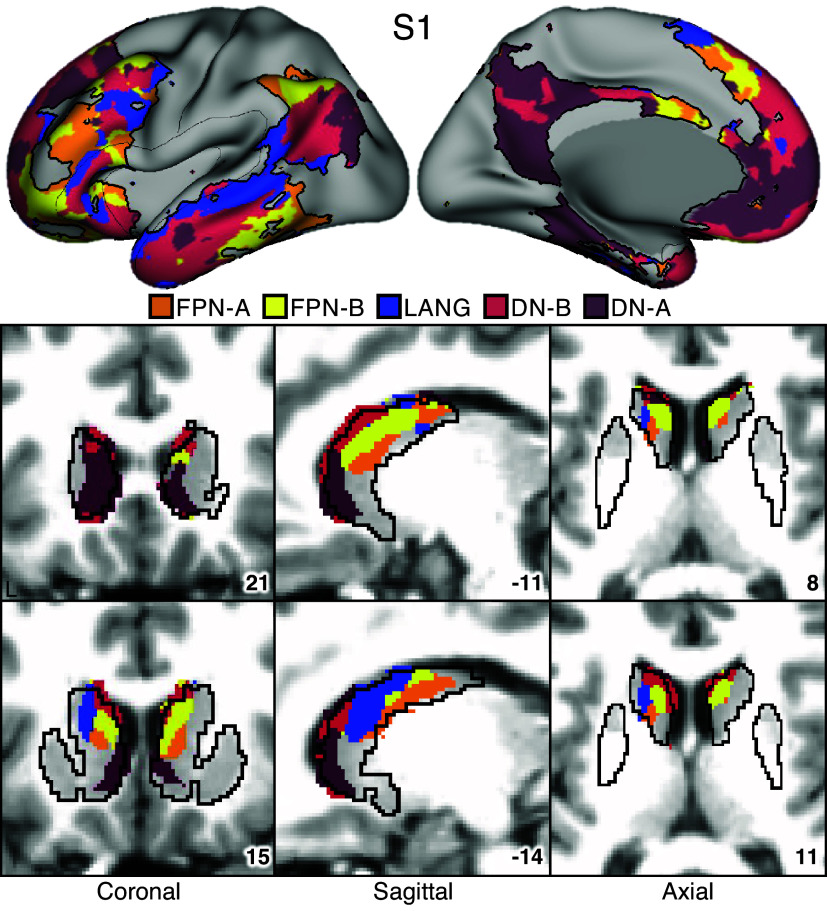
Striatal association megaclusters for *S1*. Networks within the cerebral cortex were estimated with a multisession hierarchical Bayesian model (MS-HBM) and include 5 higher-order association networks as illustrated here (19). The striatum was parcellated by assigning each voxel to its most correlated network (out of 15) in the cerebral cortex (see text). *Top*: inflated lateral and medial surfaces depict a cluster of 5 cortical networks that repeat across multiple cortical zones including posterior parietal cortex, temporal cortex, and prefrontal cortex (PFC). We refer to these repeating clusters as supra-areal association megaclusters (SAAMs). Within each SAAM, Frontoparietal Network-A (FPN-A, orange), Frontoparietal Network-B (FPN-B, yellow), Language (LANG, blue), Default Network-B (DN-B, red), and Default Network-A (DN-A, dark red) are adjacent to one another. The black outlines mark the combined borders around FPN-A, FPN-B, LANG, DN-B, and DN-A for several SAAMs. *Bottom*: multiple views of the striatum illustrate representations of the 5 association networks in the caudate. Coordinates at *bottom right* of each panel indicate the slice in the MNI152 atlas. Visualized data are from *dataset 1*. FPN-A, FPN-B, LANG, DN-B, and DN-A are observed with side-by-side relations within the caudate of both participants. These juxtapositions recapitulate the SAAMs within the cerebral cortex and are referred to as striatal association megaclusters. See Supplemental Fig. S10 for visualization of additional sections in both the discovery (*dataset 1*) and replication (*dataset 2*) datasets.

**Figure 4.**
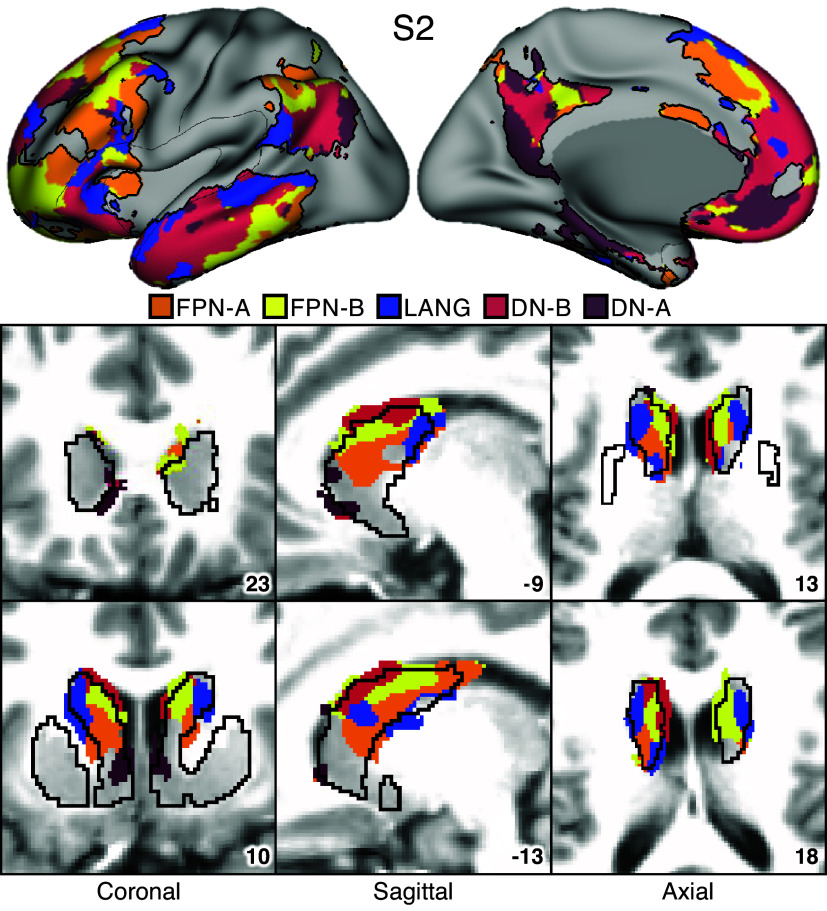
Striatal association megaclusters for *S2*. Networks within the cerebral cortex were estimated with a multisession hierarchical Bayesian model (MS-HBM) and include 5 higher-order association networks as illustrated here (19). The striatum was parcellated by assigning each voxel to its most correlated network (out of 15) in the cerebral cortex (see text). *Top*: inflated lateral and medial surfaces depict a cluster of 5 cortical networks that repeat across multiple cortical zones including posterior parietal cortex, temporal cortex, and prefrontal cortex (PFC). We refer to these repeating clusters as supra-areal association megaclusters (SAAMs). Within each SAAM, Frontoparietal Network-A (FPN-A, orange), Frontoparietal Network-B (FPN-B, yellow), Language (LANG, blue), Default Network-B (DN-B, red), and Default Network-A (DN-A, dark red) are adjacent to one another. The black outlines mark the combined borders around FPN-A, FPN-B, LANG, DN-B, and DN-A for several SAAMs. *Bottom*: multiple views of the striatum illustrate representations of the 5 association networks in the caudate. Coordinates at *bottom right* of each panel indicate the slice in the MNI152 atlas. Visualized data are from *dataset 1*. FPN-A, FPN-B, LANG, DN-B, and DN-A are observed with side-by-side relations within the caudate of both participants. These juxtapositions recapitulate the SAAMs within the cerebral cortex and are referred to as striatal association megaclusters. See Supplemental Fig. S10 for visualization of additional sections in both the discovery (*dataset 1*) and replication (*dataset 2*) datasets.

The first step in generating the striatal parcellations was to estimate the organization of networks within the cerebral cortex. Figure 3 and Figure 4 illustrate that the anatomical positions of the cortical association networks of interest for *S1* and *S2.* Du et al. (19) provide comprehensive visualizations of all 15 networks. Five networks were robustly identified (and replicated, Supplemental Fig. S9) with close juxtapositions repeatedly across the cerebral cortex. We refer to these clusters of juxtaposed cerebral networks as supra-areal association megaclusters or SAAMs (19). The putative cognitive control networks FPN-A and FPN-B were next to each other and spatially juxtaposed with the trio of domain-specialized networks, LANG, DN-B, and DN-A. Although the exact boundaries for the SAAMs varied, the spatial juxtapositions were apparent in both participants and across multiple parietal, temporal, and prefrontal association zones.

Striatal assignments to each of the five cortical association networks revealed the presence of distinct subregions in the caudate in both participants that were linked to each of the five separate networks (Fig. 3 and Fig. 4; Supplemental Fig. S10). DN-A is represented in the dorsomedial portion of the head of the caudate. DN-B surrounds DN-A but extends posteriorly from the head of the caudate through the body and into the tail. LANG is more lateral than DN-A and DN-B and crosses the anatomical boundaries of the caudate through the internal capsule and into the border of the putamen. Like DN-B, LANG begins in the head of the caudate and extends into the tail. Tightly interwoven FPN-A and FPN-B tend to be ventral to DN-B.

Thus, the first major new result of our intensive within-individual analyses was that five distinct association networks remained segregated (or partially segregated) within the caudate. Moreover, the relative spatial positions within the caudate roughly recapitulated the organization in the cortex, with FPN-A and FPN-B subregions next to one another, surrounded by subregions associated with networks LANG, DN-B, and DN-A. For both participants, the spatial pattern of results was replicated in a second, independent dataset (Supplemental Fig. S10).

We refer to the striatal zones containing clustered juxtapositions of the five association networks as striatal association megaclusters.

### Striatal Association Megaclusters Recapitulate Spatially Distinct Cerebral Networks

**Figure 5.**
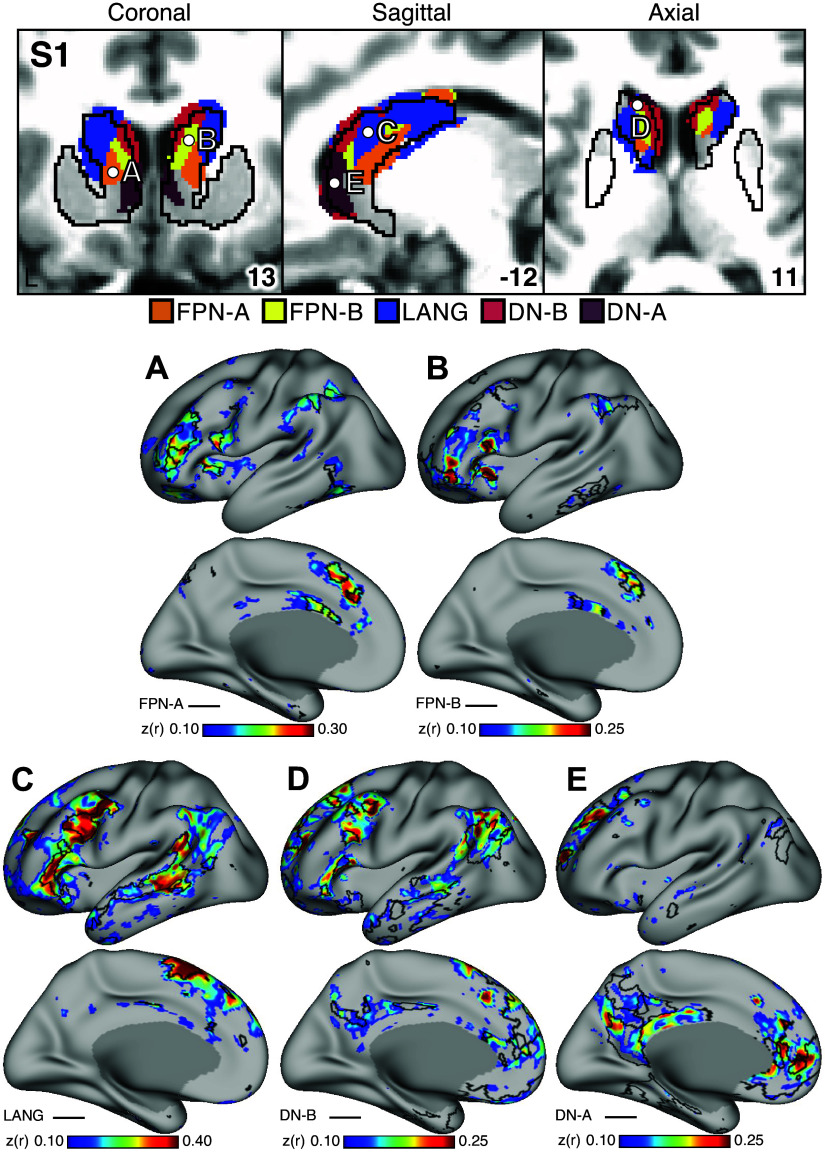
Subregions within the striatal association megaclusters are correlated with parallel distributed association networks in *S1*. To explore the specificity of subregions within the striatal association megaclusters, a model-free seed-based functional connectivity MRI (fcMRI) method was used to map cortical networks correlated with side-by-side seed regions within the caudate. In each case, the striatum-to-cortex correlation maps are from independent data that were not used to derive the striatal parcellations (i.e., *datasets 1* and *2* were used for striatal parcellations, *dataset 3* was used to estimate the striatum-to-cortex correlation maps). *Top*: the striatal subregions associated with each network are shown in the caudate (*dataset 2* used for visualization), with white filled circles illustrating the locations of the 5 separate seed regions (labeled *A–E*). *Bottom*: the seed region in the caudate assigned to Frontoparietal Network-A (FPN-A; *A*) displays spatially selective correlation with the distributed cortical FPN-A. Similarly, spatially selective cortical networks are revealed for Frontoparietal Network-B (FPN-B; *B*), Language (LANG; *C*), Default Network-B (DN-B; *D*), and Default Network-A (DN-A; *E*). The correlation maps are plotted as *z*(*r*) with the jet color scale at *bottom*. The Supplemental Materials display the patterns of striatal correlation from seed regions placed within the cortical networks (Supplemental Figs. S13–S17 for *S1* and Supplemental Figs. S18–S22 for *S2*). Coordinates at *bottom right* of each panel indicate the slice in the MNI152 atlas.

**Figure 6.**
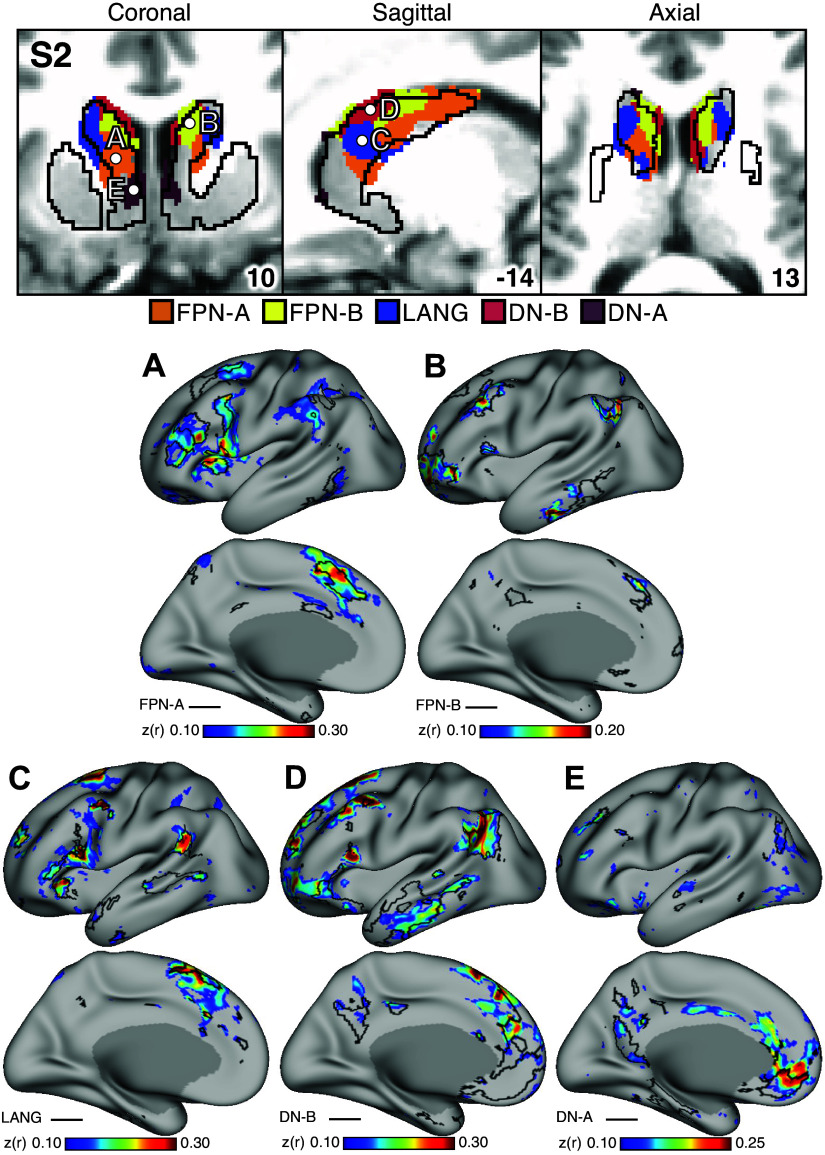
Subregions within the striatal association megaclusters are correlated with parallel distributed association networks in *S2*. To explore the specificity of subregions within the striatal association megaclusters, a model-free seed-based functional connectivity MRI (fcMRI) method was used to map cortical networks correlated with side-by-side seed regions within the caudate. In each case, the striatum-to-cortex correlation maps are from independent data that were not used to derive the striatal parcellations (i.e., *datasets 1* and *2* were used for striatal parcellations, *dataset 3* was used to estimate the striatum-to-cortex correlation maps). *Top*: the striatal subregions associated with each network are shown in the caudate (*dataset 2* used for visualization) with white filled circles illustrating the locations of the 5 separate seed regions (labeled *A–E*). *Bottom*: the seed region in the caudate assigned to Frontoparietal Network-A (FPN-A; *A*) displays spatially selective correlation with the distributed cortical FPN-A. Similarly, spatially selective cortical networks are revealed for Frontoparietal Network-B (FPN-B; *B*), Language (LANG; *C*), Default Network-B (DN-B; *D*), and Default Network-A (DN-A; *E*). The correlation maps are plotted as *z*(*r*) with the jet color scale at *bottom*. The Supplemental Materials display the patterns of striatal correlation from seed regions placed within the cortical networks (Supplemental Figs. S13–S17 for *S1* and Supplemental Figs. S18–S22 for *S2*). Coordinates at *bottom right* of each panel indicate the slice in the MNI152 atlas.

A key test of segregation of the caudate subregions was undertaken by examining whether closely juxtaposed seed regions placed within the caudate could recapitulate the spatially distinct cortical networks. In independent data for *S1* and *S2* (*dataset 3*), seed regions placed in the DN-A striatal zone from the discovery and replication parcellations (*datasets 1* and *2*) produced correlations that largely respected the boundaries of the MS-HBM-defined cortical FPN-A network (Fig. 5*A* and Fig. 6*A*). The same was true for seed regions placed in the FPN-B (Fig. 5*B* and Fig. 6*B*), LANG (Fig. 5*C* and Fig. 6*C*), DN-B (Fig. 5*D* and Fig. 6*D*), and DN-A (Fig. 5*E* and Fig. 6*E*) striatal zones. The correlation patterns do show some blur into adjacent networks. In particular, the FPN-A striatal seed regions generated correlations in the cortical extent of FPN-A and into adjacent FPN-B. Similarly, the DN-B striatal seed regions primarily recapitulated cortical DN-B, but portions of cortical LANG were also implicated.

### Cortical Signal Blurs into the Striatum

Although the replication of results within and across participants indicates that the identified patterns are robust, there is a potential confound that could cause systematic errors in validity: cortical fMRI responses can be stronger than subcortical responses. Thus, it is possible that cortical signal blur could impact the parcellation within the striatum (see Ref. 11 for discussion). This feature affects the multiple prior studies of striatal organization using human neuroimaging approaches and the present work. To test the impact of spatial blur from adjacent cortical regions, we used a winner-takes-all strategy on smoothed (4-mm FWHM Gaussian kernel) and unsmoothed data, where correlation values below a specific threshold were excluded. We systematically raised thresholds to observe whether any voxel assignments in the striatum were continuations of cerebral correlation patterns. Supplemental Figs. S11 and S12 illustrate the results and indicate that signal blur from cortex may be impacting some striatal assignments.

Estimates in the lateral striatum, which borders insular cortex, and the ventral striatum, which borders orbitofrontal cortex, are ambiguous because of cortical signal bleed. Striatal association megaclusters in the caudate were largely (and clearly) separate from possible signal blur from adjacent cortex. One relevant striatal assignment of potential concern is the DN-A/DN-B cortical network portions that fall along the midline and could potentially extend into the striatum (see Supplemental Figs. S11 and S12 as well as the correlation maps in Supplemental Figs. S17 and S22). Thus, the location of the DN-A assignment in the caudate might be refined as higher-resolution data are interrogated. Furthermore, there are regions in more ventral portions of the striatum, which are not the focus of our results but were emphasized in our prior parcellations (11), that may be strongly affected by spatial blur given the proximity of the ventral regions of the striatum and the midline cortical frontal regions involved in DN-A and DN-B. Critically, the caudate zones associated with FPN-A, FPN-B, and LANG are particularly distinct by every measure (e.g., see the clear differences in even the raw correlations patterns in Supplemental Figs. S17–S22) and discontinuous with any nearby cortical surface network assignments (Supplemental Figs. S11 and S12).

### Prospective Replication of Striatal Organization in 15 Novel Participants

**Figure 7.**
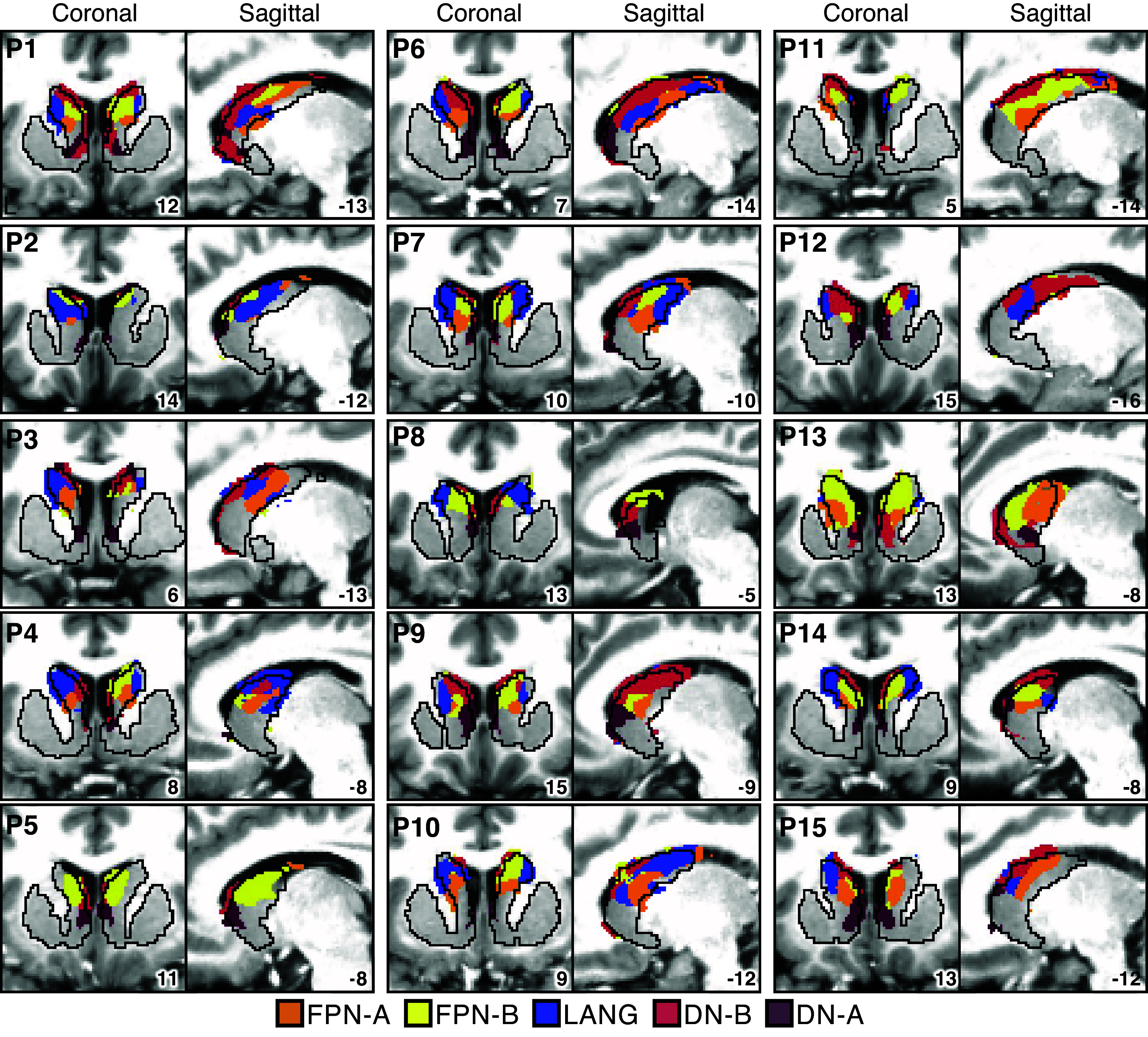
Striatal association megaclusters prospectively replicate across new participants. Sagittal and coronal slices of the striatum illustrate representations of the 5 higher-order association networks in the caudate for *P1–P15*. Frontoparietal Network-A (FPN-A), Frontoparietal Network-B (FPN-B), Language (LANG), Default Network-B (DN-B), and Default Network-A (DN-A) are observed again with side-by-side relations that fall within the caudate. These juxtapositions replicate the striatal association megaclusters observed in *S1* and *S2* (Figs. 3 and 4). Coordinates at *bottom right* of each panel indicate the slice in the MNI152 atlas.

FPN-A, FPN-B, LANG, DN-B, and DN-A have representations that are localized to the caudate for both *S1* and *S2*, with variability in the specific locations and boundaries of the subregions. To determine whether the presence of striatal association megaclusters generalizes to additional individuals, we explored the striatal parcellations in 15 novel participants (*P1–P15*), using the same winner-takes-all strategy described above. Although there was considerable spatial variability between individuals, in all but one participant we found closely juxtaposed assignments to FPN-A, FPN-B, LANG, DN-B, and DN-A in the caudate (Fig. 7). The one clear failure of generalization was that *P5* did not have any voxels assigned to LANG in the left hemisphere of the caudate. Overall, the caudate parcellation results for *P1–P15* were similar to those for *S1* and *S2*, suggesting that the presence of striatal association megaclusters is a finding that generalizes across many individuals.

### Laterality in the Striatum Mimics Laterality in the Cerebral Cortex

**Figure 8.**
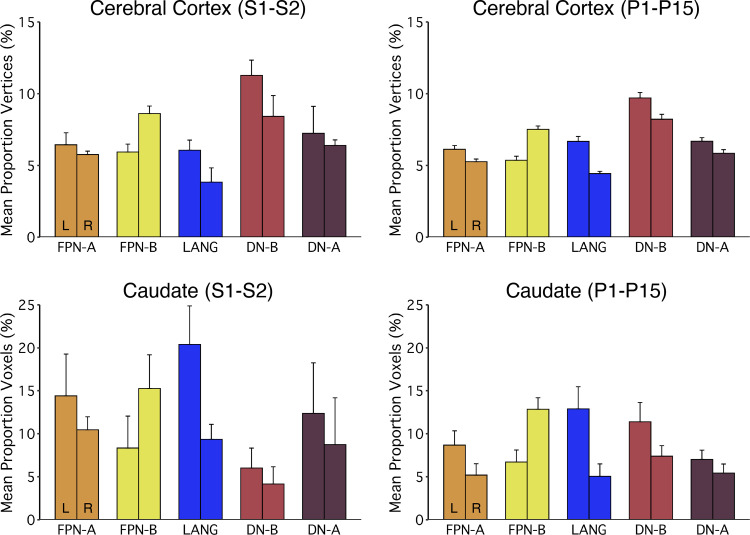
Laterality of striatal association regions parallels the laterality of networks in the cerebral cortex. *Top*: the mean proportion of vertices assigned to Frontoparietal Network-A (FPN-A), Frontoparietal Network-B (FPN-B), Language (LANG), Default Network-B (DN-B), and Default Network-A (DN-A) in the left (L) and right (R) cortical hemispheres for *S1* and *S2* (*left*) and for *P1–P15* (*right*). Note that the LANG network is left lateralized and FPN-B is right lateralized [replicating Braga et al. (44)]. *Bottom*: the proportion of voxels in the striatum assigned to FPN-A, FPN-B, LANG, DN-B, and DN-A in the left (L) and right (R) hemispheres for *S1* and *S2* (*left*) and for *P1–P15* (*right*). Note that the patterns of laterality in the caudate for both samples recapitulate the laterality of the cerebral cortex. Error bars indicate SE.

In both groups of participants, we observed lateralization of networks in the cerebral cortex that are consistent with previous reports (20). In particular, the LANG network is left lateralized and FPN-B^2^ is right lateralized (Fig. 8, *top*). In both groups of participants, the striatal lateralization mimicked the laterality found in the cerebral cortex (Fig. 8, *bottom*). Importantly, correlation values between the striatum and both hemispheres of the cerebral cortex were used for assigning voxels to a particular network, so the lateralization was not biased by cortical asymmetries or assumptions.

## DISCUSSION

The basal ganglia operate on inputs to the striatum from diverse cortical regions with segregation between motor, affective, and cognitive loops (Ref. 5; see also Ref. 45). Here, using within-individual precision mapping, we discovered clustered representations of five separate higher-order association networks with side-by-side juxtapositions in the caudate. By placing separate seed regions within the five identified subregions of the caudate, we recapitulated the topography of the distinct cortical networks within each participant, supporting the inference that they participate in segregated or partially segregated networks. These results have conceptual implications for understanding striatal organization and function, as well as practical implications for the experimental study of the basal ganglia.

### Striatal Association Megaclusters

Separate representations of the five higher-order association networks were found within the caudate of both participants in the initial analyses (*S1* and *S2*) and in 14 of 15 participants in the generalization sample (*P1–P15*). We refer to these as striatal association megaclusters because of their similarity to SAAMs present in the cerebral cortex, as reported by Du et al. (19). Within the cerebral cortex, SAAMs are characterized by the juxtaposition of FPN-A, FPN-B, LANG, DN-B, and DN-A in a repeating fashion across parietal, temporal, and prefrontal zones. In the striatum, although there are idiosyncratic details that vary between participants, there are similar spatial relations among the five networks. DN-A is found in the dorsomedial portion of the head of the caudate, with DN-B surrounding it and extending posteriorly through the body and into the tail. LANG is more lateral, beginning in the head and extending into the anterior portion of the tail, whereas FPN-A and FPN-B are interdigitated and ventral to DN-B. The relative spatial positions within the caudate recapitulate cortical organization, with FPN-A and FPN-B regions next to one another, surrounded by regions associated with networks LANG, DN-B, and DN-A. Importantly, this organization was not obligated by the analysis, as voxels could be assigned to any 1 of the 15 cortical networks.

Prior work has suggested that multiple distinct networks remain segregated in the caudate. For example, in Choi et al.’s (11) analysis of group-averaged data in 1,000 individuals, distinct regions in the caudate were associated with a putative control network separate from the default network. However, further functional-anatomical details could not be resolved, including identification of regions in the caudate linked to language. In Greene et al.’s (18) pioneering work examining the striatum within individuals, they noted network-specific caudate regions linked to what is here termed FPN-A as well as the default network (see also Ref. 13). Within their supplementary figures, they also provided evidence that some individuals possess a caudate representation of the LANG network.^3^ What is novel in the present work is the full mapping of five distinct networks that maintain segregation within the caudate. Distinct caudate regions linked to language and social functions could be consistently identified that were spatially distinct from other regions linked to cognitive control.

As can also be appreciated in Fig. 7, there was substantial variability across individuals in the size and boundaries of the regions, with some individuals possessing a greater representation of certain networks over others. We are hesitant to interpret this variability given that it is likely a combination of technical limitations of fMRI and idiosyncratic anatomical differences between individuals. What is striking is that in almost every individual all five networks could be identified with roughly the same relative positions within the caudate, suggesting that segregation is maintained in the striatum and further that the relative positions of the network-specific regions are conserved.

The presence of striatal association megaclusters is consistent with the long-held idea that the basal ganglia possess multiple closed-loop circuits that operate on segregated channels of inputs (5). Here, we revealed an unexpected level of specificity that segregates higher-order association networks within the caudate itself. This extension is surprising given prior neuroimaging studies (e.g., our own earlier work in Ref. 11) and anatomical tract tracing studies that suggest convergence within the caudate (9). Our ability to detect and replicate spatially segregated subregions of the caudate is enabled by the precision within-individual approach adopted.

### Striatal Organization Parallels Cerebral Cortex and Cerebellar Organization

Following the advent of fcMRI to estimate cortical networks (48), within-individual precision methods have increased our understanding of the details of cortical organization (e.g., Refs. 19, 20, 44, 49–55; see also Refs. 46, 47, 56). Specifically, networks that initially appeared to involve large, less specialized regions were discovered in several instances to possess multiple tightly juxtaposed regions that are blurred together by group averaging methods (52). For example, with precision approaches the extensively studied “default network” was found to be a combination of multiple, parallel networks with functionally dissociable side-by-side regions (19, 44, 49, 57) (see Refs. 58, 59 for discussion). In a recent discovery, precision mapping revealed the existence of intereffector regions in the precentral gyrus that are distinct from the canonical motor effector representations in M1 (51). Precision mapping of the striatum, as reported here, similarly finds evidence for segregation in the caudate where prior work, including our own, found convergence (6, 11).

In this light, it is interesting to emphasize that the striatal association megaclusters parallel the complex yet elegant organization of cerebral and cerebellar cortical association zones. In the cerebral cortex, multiple SAAMs with the same motif of network juxtapositions are present consistently across individuals (19). The cerebellum possesses a parallel organization, an association megacluster, in at least one large zone. Specifically, in the cerebellum there is a consistent clustering of the five networks around Crus I/II (see Fig. 10 in Ref. 21 and Ref. 60). Furthermore, Marek et al. (61) used a distinct set of network estimates and revealed a similar set of side-by-side juxtapositions in Crus I/II including distinctions between regions linked to FPN and DN. Thus, as precision mapping approaches have been applied, greater spatial specificity and segregation have been observed in both cerebral and cerebellar cortices. Our understanding of striatal organization has similarly evolved with the application of within-individual precision methods (13, 18).

The present findings are consistent with a framework in which the basal ganglia and cerebellum are nodes in an integrated circuit with the cerebral cortex and furthermore that the integration involves separation of multiple functional domains (62). The repeating motif we find here in the striatum, and that was previously observed in the cerebellum, suggests that basal ganglia-cerebellar-cerebral cortical networks maintain segregated, or partially segregated, channels across multiple higher-order functional domains. This segregation forms megaclusters in the caudate that echo their counterparts in the cerebral cortex including in their laterality (Fig. 8).

### Relevance to Anatomical Projection Patterns Observed in Nonhuman Primates

The present results may be relevant to interpretation of certain puzzling macroorganizational features of anatomical projection patterns observed in nonhuman primates. Yeterian and Van Hoesen (4) noted diverse projections to the caudate from distinct regions of association cortex. Comparing patterns across injections, they hypothesized that cortical regions that are connected to one another also share projection territories in the striatum, consistent with the idea that they are nodes in distributed association networks (63, 64). Selemon and Goldman-Rakic (3) examined, in detail, paired injections across PFC and parietal association zones that showed some overlap but also, for certain injection pairs, clear separation in the caudate. They suggested that the connected cortical regions may not always converge on the same caudate zones but rather be adjacent to one another (see also Ref. 65). The present results are informative as to why such diversity in projection patterns might arise.

The cortical association zones that possess distinct side-by-side regions are anatomically variable from one person to the next. The motif and relative positions are similar, but the absolute positions of network regions on the cortical surface are highly idiosyncratic (19). If similar features are present in nonhuman primates, variable combinations of networks would be sampled from injection to injection. Consistent with this possibility, tracer injections across cases and association zones reveal label in the caudate that sometimes contains considerable macroorganizational overlap and in other instances side-by-side adjacencies (3, 4). Such patterns are expected if the injections are, by happenstance, hitting variable combinations of adjacent cortical networks. It will be interesting, in the future, to chart whether comprehensive direct anatomical estimates in nonhuman primates converge with the indirect estimates reported here.

### Limitations and Open Questions

Here we relied on fcMRI analyses to infer network structure within the cerebral cortex and its relations to the striatum. There are a variety of caveats to such methods and their interpretation (21, 66–71). One specific limitation for estimating striatal organization is signal bleed from the cortex to the striatum. Voxels in the striatum that are spatially near insular and orbitofrontal cortices are the most likely to have corrupted signals impacted by stronger cortical signals. The striatum generally possesses lower SNR than the cortex. Therefore, it is important to carefully inspect striatal patterns in relation to nearby cortex. Supplemental Figs. S11, S12, S17, and S22 illustrate this challenge and reveal potential artifacts of cortical blur into the striatum in several zones that are not the focus of the present work (near to the ventral striatum in particular). Assignments to DN-A along the midline are potentially of concern and may require further refinement, a possibility that will likely require examination at a higher resolution and field strength (7 T or beyond).

A similar limitation in analysis of functional-anatomical details relates to interpretation of overlap within the striatum itself. Although our control analyses of striatal motor zones established that the present methods could differentiate distinct zones (Fig. 2), spatial blur is nonetheless expected and remains a challenge. In this light, Greene and colleagues (18) note a region of overlap in the caudate that includes contributions of multiple higher-order association networks (see their Fig. 5B). We too note regions of overlap consistent with their observations. However, given that there is also clear evidence of network-specific regions in the caudate near these zones of overlap, we conservatively leave open the question of whether the overlap reflects anatomical integration or is a confounding effect of spatial blur. Examination at higher resolution and field strength may assist in exploring the question of segregation versus convergence.

Another limitation of the presented research is that we did not characterize the function of caudate subregions with task-based data or comprehensively map all networks across the stratum including the ventral striatum. In the cerebral cortex, task-based fMRI has expanded our understanding of distinct functions associated with each network. For example, FPN-A is engaged for domain-flexible tasks that are cognitively demanding (e.g., Refs. 19, 72; see also Ref. 73), whereas LANG, DN-B, and DN-A are domain specific (19, 57, 74). Here, although we demonstrate the functional-anatomical relations between specific cortical networks and subregions of the caudate, we do not test how these subregions respond to various cognitive task demands. Further research will be necessary to characterize caudate function with a careful consideration of detailed anatomical organization. Such research may require methods that increase resolution while maintaining high SNR. Analyses that seek to comprehensively explore the striatum will benefit from acquisition protocols that minimize susceptibility artifacts that can particularly affect the SNR of the ventral striatum (see Fig. 1).

**Figure 9.**
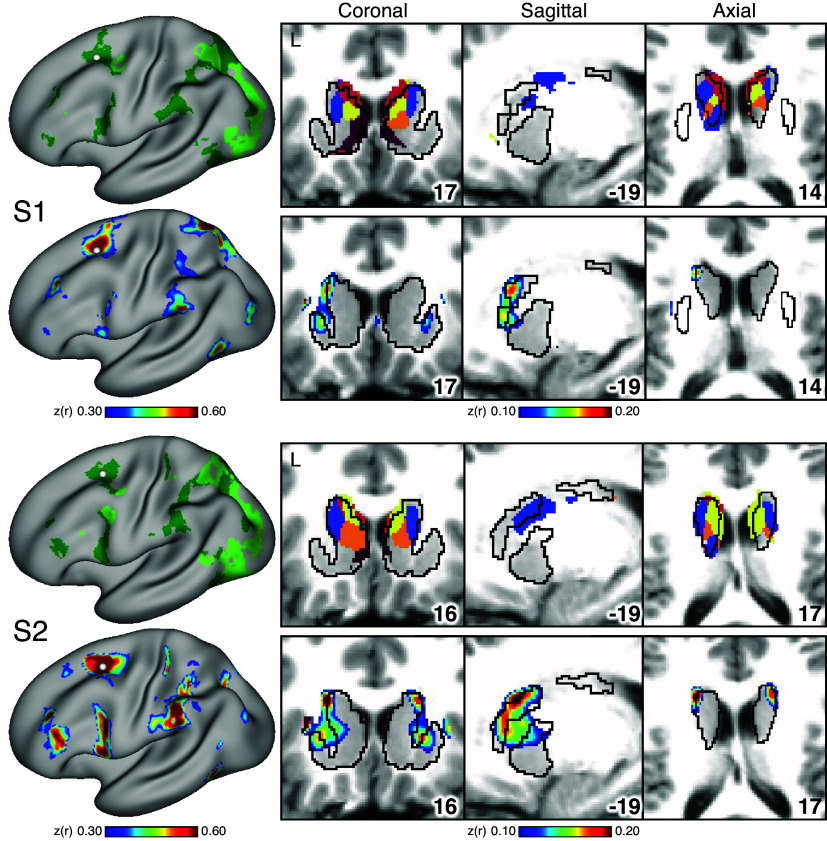
Seed regions in Dorsal Attention Network-A (dATN-A) correlate with a striatal region lateral to the striatal association megaclusters. The frontal eye field (FEF) is a cortical region that projects to the caudate in macaques. To test whether a candidate human homolog has correlations in the striatum that overlap with (or are distinct from) the striatal association megaclusters, we conducted a pilot analysis by placing a seed region in the approximate location of putative human FEF. Specifically, we used the dATN-A region situated anterior to the precentral gyrus to guide seed region placement for each participant. Then, we visualized correlations relative to the striatal association megaclusters. The *top* row for each participant shows the cortical parcellation of dATN-A (light green) and Dorsal Attention Network-B (dATN-B, dark green) on the *left* and the striatal association megaclusters on the *right*. The seed region in dATN-A is visualized with a white circle. The *bottom* row for each participant shows the correlations with the seed region (at or near putative FEF) on the cortical surface (*left*) and in the striatum (*right*). Note that the correlations fall within regions of the caudate adjacent to the regions associated with the striatum association megaclusters. The correlation maps are plotted as *z*(*r*) with the color scale at *bottom*. Coordinates on the *bottom right* of each panel indicate the slice in the MNI152 atlas.

It is also important to note that our focus on five specific networks linked to the caudate does not negate the possibility that other cortical regions also project to the caudate. For example, extensive work with nonhuman primates indicates that the frontal eye field (FEF) projects to the caudate (75–77) and that the nearby supplementary eye field (SEF) has a different pattern of projections to a nearby region of the caudate (77, 78). In preliminary analyses, we found a putative FEF-correlated region in the caudate that is spatially adjacent to the striatal association megaclusters (Fig. 9). Future work should acquire data to functionally localize FEF and SEF and measure how they are coupled with the striatum in humans along with SAAMs.

Although we provide evidence for segregated or partially segregated regions, in terms of the macroorganization of the caudate, our findings do not imply that interactions are absent or that our methods are able to observe all local circuit features. In addition to reports of overlap across networks in human functional neuroimaging data, several direct anatomical observations also highlight complexities by which anatomical organization in the stratum supports convergent versus anatomically segregated circuitry. For example, the dual injection cases of Selemon and Goldman-Rakic (3) revealed that distinct cortical regions project to interdigitated terminal fields within the striatum. This feature was particularly prominent in their Case 18 and suggests an anatomical feature of striatal organization that is beyond the present resolution of fMRI. Relatedly, some hypotheses about integration in the basal ganglia focus on how projection zones asymmetrically extend across anatomical domains. For example, Haber, Fudge, and McFarland (79) examined projections to and from the midbrain using an extensive series of nonhuman primate tracer injections. They observed a hierarchy by which projections originating from the shell of the NAc extend into the loops of adjacent parallel circuits, forming a cascading influence on motor circuits (see also Ref. 80). Our analyses of macroorganizational patterns in the striatum would not be able to detect such anatomical features.

### Conclusions

We describe a new parcellation of the striatum that considers the idiosyncratic anatomical differences between people. In so doing, we observed striatal association megaclusters of tightly juxtaposed zones of the caudate that are coupled with higher-order association networks FPN-A, FPN-B, LANG, DN-B, and DN-A. These results extend the general notion of parallel specialized basal ganglia circuits (5) with the additional discovery that there is fine-grained separation of multiple distinct networks, even within the caudate.

## DATA AVAILABILITY

 All data are publicly available on NIMH Data Archive (NDA); striatal parcellations are available on BALSA (https://balsa.wustl.edu/); data for Fig. 8 and all code are available on Open Science Framework (OSF; https://osf.io/rhbfn/).

## SUPPLEMENTAL MATERIALS

10.6084/m9.figshare.25229849 Supplemental Figs. S1–S22: https://doi.org/10.6084/m9.figshare.25229849.

## GRANTS

This work was supported by NIH Grant MH124004, NIH Shared Instrumentation Grant S10OD020039, and National Science Foundation (NSF) Grant DRL2024462. H.L.K. was supported by NIH F99/K00 Grant 8K00DA058542.

## DISCLOSURES

No conflicts of interest, financial or otherwise, are declared by the authors.

## AUTHOR CONTRIBUTIONS

H.L.K. and R.L.B. conceived and designed research; H.L.K. and M.C.E. performed experiments; H.L.K., N.S.-G., and J.D. analyzed data; H.L.K. and R.L.B. interpreted results of experiments; H.L.K. prepared figures; H.L.K. drafted manuscript; H.L.K. and R.L.B. edited and revised manuscript; H.L.K., N.S.-G., J.D., M.C.E., and R.L.B. approved final version of manuscript.

## Footnotes

1 A challenge in using masks from T1w structural data is that the BOLD data are lower resolution and are smoothed, causing the functional response to be spatially more extensive. The dilated mask allows the full extent of the correlation patterns and parcellation to be observed.

2 The naming conventions for FPN-A and FPN-B are inconsistent between Du et al. (19) and Braga et al. (20) such that FPN-A and FPN-B are flipped between the two papers. Thus, the network labeled FPN-B here [and in Du et al. (19)] corresponds to FPN-A from Braga et al. (20). The network labeled FPN-A here corresponds to FPN-B from Braga et al. (20).

3 What is termed here the LANG network based on Ref. 19 is labeled as the ventral attention network (VAN) in Greene et al. (18). We label the network in relation to language function because of its identification in meaning-based sentence processing tasks (19, 44, 46, 47), its left lateralization (Fig. 8), and evidence that it can be distinguished from other nearby networks associated with cognitive control and attention (19, 44).

## References

[1] Glees P. The anatomical basis of cortico-striate connexions. J Anat 78: 47–51, 1944. 17104940 PMC1272574

[2] Goldman PS, Nauta WJ. An intricately patterned prefronto-caudate projection in the rhesus monkey. J Comp Neurol 72: 369–386, 1977. 401838 10.1002/cne.901710305

[3] Selemon LD, Goldman-Rakic PS. Longitudinal topography and interdigitation of corticostriatal projections in the rhesus monkey. J Neurosci 5: 776–794, 1985. doi:10.1523/JNEUROSCI.05-03-00776.1985. 2983048 PMC6565017

[4] Yeterian EH, Van Hoesen GW. Cortico-striate projections in the rhesus monkey: the organization of certain cortico-caudate connections. Brain Res 139: 43–63, 1978. doi:10.1016/0006-8993(78)90059-8. 413609

[5] Alexander GE, DeLong MR, Strick PL. Parallel organization of functionally segregated circuits linking basal ganglia and cortex. Annu Rev Neurosci 9: 357–381, 1986. doi:10.1146/annurev.ne.09.030186.002041. 3085570

[6] Choi EY, Tanimura Y, Vage PR, Yates EH, Haber SN. Convergence of prefrontal and parietal anatomical projections in a connectional hub in the striatum. NeuroImage 146: 821–832, 2017. doi:10.1016/j.neuroimage.2016.09.037. 27646127 PMC5841917

[7] Ferry AT, Öngür D, An X, Price JL. Prefrontal cortical projections to the striatum in macaque monkeys: evidence for an organization related to prefrontal networks. J Comp Neurol 425: 447–470, 2000. doi:10.1002/1096-9861(20000925)425:3<447::AID-CNE9>3.0.CO;2-V. 10972944

[8] Korponay C, Choi EY, Haber SN. Corticostriatal projections of macaque area 44. Cereb Cortex Commun 1: tgaa079, 2020. doi:10.1093/texcom/tgaa079. 33283184 PMC7699020

[9] Averbeck BB, Lehman J, Jacobson M, Haber SN. Estimates of projection overlap and zones of convergence within frontal-striatal circuits. J Neurosci 34: 9497–9505, 2014. doi:10.1523/JNEUROSCI.5806-12.2014. 25031393 PMC4099536

[10] Barnes KA, Cohen AL, Power JD, Nelson SM, Dosenbach YB, Miezin FM, Petersen SE, Schlaggar BL. Identifying basal ganglia divisions in individuals using resting-state functional connectivity MRI. Front Syst Neurosci 4: 18, 2010. doi:10.3389/fnsys.2010.00018. 20589235 PMC2892946

[11] Choi EY, Yeo BT, Buckner RL. The organization of the human striatum estimated by intrinsic functional connectivity. J Neurophysiol 108: 2242–2263, 2012. doi:10.1152/jn.00270.2012. 22832566 PMC3545026

[12] Di Martino A, Scheres A, Margulies DS, Kelly AM, Uddin LQ, Shehzad Z, Biswal B, Walters JR, Castellanos FX, Milham MP. Functional connectivity of human striatum: a resting state fMRI study. Cereb Cortex 18: 2735–2747, 2008. doi:10.1093/cercor/bhn041. 18400794

[13] Gordon EM, Laumann TO, Marek S, Newbold DJ, Hampton JM, Seider NA, Montez DF, Nielsen AM, Van AN, Zheng A, Miller R, Siegel JS, Kay BP, Snyder AZ, Greene DJ, Schlaggar BL, Petersen SE, Nelson SM, Dosenbach NU. Individualized functional subnetworks connect human striatum and frontal cortex. Cereb Cortex 32: 2868–2884, 2022. doi:10.1093/cercor/bhab387. 34718460 PMC9247416

[14] Greene DJ, Laumann TO, Dubis JW, Ihnen SK, Neta M, Power JD, Pruett JR, Black KJ, Schlaggar BL. Developmental changes in the organization of functional connections between the basal ganglia and cerebral cortex. J Neurosci 34: 5842–5854, 2014. doi:10.1523/JNEUROSCI.3069-13.2014. 24760844 PMC3996213

[15] Jarbo K, Verstynen TD. Converging structural and functional connectivity of orbitofrontal, dorsolateral prefrontal, and posterior parietal cortex in the human striatum. J Neurosci 35: 3865–3878, 2015. doi:10.1523/JNEUROSCI.2636-14.2015. 25740516 PMC4461697

[16] Marquand AF, Haak KV, Beckmann CF. Functional corticostriatal connection topographies predict goal-directed behaviour in humans. Nat Hum Behav 1: 0146, 2017. doi:10.1038/s41562-017-0146. 28804783 PMC5549843

[17] O’Rawe JF, Leung HC. Topographic organization of the human caudate functional connectivity and age-related changes with resting-state fMRI. Front Syst Neurosci 16: 966433, 2022. doi:10.3389/fnsys.2022.966433. 36211593 PMC9543452

[18] Greene DJ, Marek S, Gordon EM, Siegel JS, Gratton C, Laumann TO, Gilmore AW, Berg JJ, Nguyen AL, Dierker D, Van AN, Ortega M, Newbold DJ, Hampton JM, Nielsen AN, McDermott KB, Roland JL, Norris SA, Nelson SM, Snyder AZ, Schlaggar BL, Petersen SE, Dosenbach NU. Integrative and network-specific connectivity of the basal ganglia and thalamus defined in individuals. Neuron 105: 742–758.e6, 2020. doi:10.1016/j.neuron.2019.11.012. 31836321 PMC7035165

[19] Du J, DiNicola LM, Angeli PA, Saadon-Grosman N, Sun W, Kaiser S, Ladopoulou J, Xue A, Yeo BT, Eldaief MC, Buckner RL. Organization of the human cerebral cortex estimated within individuals: networks, global topography, and function. *J Neurophysiol*, 2024. doi: 10.1152/jn.00308.2023. 38489238 PMC11383390

[20] Braga RM, Van Dijk KR, Polimeni JR, Eldaief MC, Buckner RL. Parallel distributed networks resolved at high resolution reveal close juxtaposition of distinct regions. J Neurophysiol 121: 1513–1534, 2019. doi:10.1152/jn.00808.2018. 30785825 PMC6485740

[21] Xue A, Kong R, Yang Q, Eldaief MC, Angeli PA, DiNicola LM, Braga RM, Buckner RL, Yeo BT. The detailed organization of the human cerebellum estimated by intrinsic functional connectivity within the individual. J Neurophysiol 125: 358–384, 2021. doi:10.1152/jn.00561.2020. 33427596 PMC7948146

[22] Saadon-Grosman N, Angeli PA, DiNicola LM, Buckner RL. A third somatomotor representation in the human cerebellum. J Neurophysiol 128: 1051–1073, 2022. doi:10.1152/jn.00165.2022. 36130164 PMC9576182

[23] Kwong KK, Belliveau JW, Chesler DA, Goldberg IE, Weisskoff RM, Poncelet BP, Kennedy DN, Hoppel BE, Cohen MS, Turner R. Dynamic magnetic resonance imaging of human brain activity during primary sensory stimulation. Proc Natl Acad Sci USA 89: 5675–5679, 1992. doi:10.1073/pnas.89.12.5675. 1608978 PMC49355

[24] Ogawa S, Tank DW, Menon R, Ellermann JM, Kim SG, Merkle H, Ugurbil K. Intrinsic signal changes accompanying sensory stimulation: functional brain mapping with magnetic resonance imaging. Proc Natl Acad Sci USA 89: 5951–5955, 1992. doi:10.1073/pnas.89.13.5951. 1631079 PMC402116

[25] Van Essen DC, Smith SM, Barch DM, Behrens TE, Yacoub E, Ugurbil K; WU-Minn HCP Consortium. The WU-Minn Human Connectome Project: an overview. NeuroImage 80: 62–79, 2013. doi:10.1016/j.neuroimage.2013.05.041. 23684880 PMC3724347

[26] Xu J, Moeller S, Strupp J, Auerbach EJ, Chen L, Feinberg DA, Ugurbil K, Yacoub E. Highly accelerated whole brain imaging using aligned-blipped-controlled-aliasing multiband EPI (Abstract). Proc Int Soc Magn Reson Med 20: 2306, 2012.

[27] Xu J, Moeller S, Auerbach EJ, Strupp J, Smith SM, Feinberg DA, Yacoub E, Uğurbil K. Evaluation of slice accelerations using multiband echo planar imaging at 3T. NeuroImage 83: 991–1001, 2013. doi:10.1016/j.neuroimage.2013.07.055. 23899722 PMC3815955

[28] Setsompop K, Gagoski BA, Polimeni JR, Witzel T, Wedeen VJ, Wald LL. Blipped-controlled aliasing in parallel imaging for simultaneous multislice echo planar imaging with reduced g-factor penalty. Magn Reson Med 67: 1210–1224, 2012. doi:10.1002/mrm.23097. 21858868 PMC3323676

[29] van der Kouwe AJ, Benner T, Salat DH, Fischl B. Brain morphometry with multiecho MPRAGE. NeuroImage 40: 559–569, 2008. doi:10.1016/j.neuroimage.2007.12.025. 18242102 PMC2408694

[30] Mennes M, Jenkinson M, Valabregue R, Buitelaar JK, Beckmann C, Smith S. Optimizing full-brain coverage in human brain MRI through population distributions of brain size. NeuroImage 98: 513–520, 2014. doi:10.1016/j.neuroimage.2014.04.030. 24747737

[31] Weiskopf N, Hutton C, Josephs O, Deichmann R. Optimal EPI parameters for reduction of susceptibility-induced BOLD sensitivity losses: a whole-brain analysis at 3 T and 1.5 T. NeuroImage 33: 493–504, 2006. doi:10.1016/j.neuroimage.2006.07.029. 16959495

[32] Wall MB. Multiband acquisition sequences for fMRI: proceed with caution. Aperture Neuro 2023: 3, 2023. doi:10.52294/001c.91292.

[33] Fischl B. FreeSurfer. NeuroImage 62: 774–781, 2012. doi:10.1016/j.neuroimage.2012.01.021. 22248573 PMC3685476

[34] Fischl B, Sereno MI, Dale AM. Cortical surface-based analysis: II: Inflation, flattening, and a surface-based coordinate system. NeuroImage 9: 195–207, 1999. doi:10.1006/nimg.1998.0396. 9931269

[35] Cox RW. AFNI: software for analysis and visualization of functional magnetic resonance neuroimages. Comput Biomed Res 29: 162–173, 1996. doi:10.1006/cbmr.1996.0014. 8812068

[36] Cox RW. AFNI: what a long strange trip it’s been. NeuroImage 62: 743–747, 2012. doi:10.1016/j.neuroimage.2011.08.056. 21889996 PMC3246532

[37] Ojemann JG, Akbudak E, Snyder AZ, McKinstry RC, Raichle ME, Conturo TE. Anatomic localization and quantitative analysis of gradient refocused echo-planar fMRI susceptibility artifacts. NeuroImage 6: 156–167, 1997. doi:10.1006/nimg.1997.0289. 9344820

[38] Glasser MF, Sotiropoulos SN, Wilson JA, Coalson TS, Fischl B, Andersson JL, Xu J, Jbabdi S, Webster M, Polimeni JR, Van Essen DC, Jenkinson M; WU-Minn HCP Consortium. The minimal preprocessing pipelines for the Human Connectome Project. NeuroImage 80: 105–124, 2013. doi:10.1016/j.neuroimage.2013.04.127. 23668970 PMC3720813

[39] Marcus DS, Harwell J, Olsen T, Hodge M, Glasser MF, Prior F, Jenkinson M, Laumann T, Curtiss SW, Van Essen DC. Informatics and data mining tools and strategies for the Human Connectome Project. Front Neuroinform 5: 4, 2011. doi:10.3389/fninf.2011.00004. 21743807 PMC3127103

[40] Flaherty AW, Graybiel AM. Input-output organization of the sensorimotor striatum in the squirrel monkey. J Neurosci 14: 599–610, 1994. doi:10.1523/JNEUROSCI.14-02-00599.1994. 7507981 PMC6576827

[41] Flaherty AW, Graybiel AM. Two input systems for body representations in the primate striatal matrix: experimental evidence in the squirrel monkey. J Neurosci 13: 1120–1137, 1993. doi:10.1523/JNEUROSCI.13-03-01120.1993. 7680067 PMC6576612

[42] Kong R, Li J, Orban C, Sabuncu MR, Liu H, Schaefer A, Sun N, Zuo XN, Holmes AJ, Eickhoff SB, Yeo BT. Spatial topography of individual-specific cortical networks predicts human cognition, personality, and emotion. Cereb Cortex 29: 2533–2551, 2019 [Erratum in Cereb Cortex 31: 3974, 2021]. doi:10.1093/cercor/bhy123. 29878084 PMC6519695

[43] Yeo BT, Krienen FM, Sepulcre J, Sabuncu MR, Lashkari D, Hollinshead M, Roffman JL, Smoller JW, Zöllei L, Polimeni JR, Fischl B, Liu H, Buckner RL. The organization of the human cerebral cortex estimated by intrinsic functional connectivity. J Neurophysiol 106: 1125–1165, 2011. doi:10.1152/jn.00338.2011. 21653723 PMC3174820

[44] Braga RM, DiNicola LM, Becker HC, Buckner RL. Situating the left-lateralized language network in the broader organization of multiple specialized large-scale distributed networks. J Neurophysiol 124: 1415–1448, 2020. doi:10.1152/jn.00753.2019. 32965153 PMC8356783

[45] Haber SN. The primate basal ganglia: parallel and integrative networks. J Chem Neuroanat 26: 317–330, 2003. doi:10.1016/j.jchemneu.2003.10.003. 14729134

[46] Fedorenko E, Hsieh PJ, Nieto-Castañón A, Whitfield-Gabrieli S, Kanwisher N. New method for fMRI investigations of language: defining ROIs functionally in individual subjects. J Neurophysiol 104: 1177–1194, 2010. doi:10.1152/jn.00032.2010. 20410363 PMC2934923

[47] Fedorenko E, Behr MK, Kanwisher N. Functional specificity for high-level linguistic processing in the human brain. Proc Natl Acad Sci USA 108: 16428–16433, 2011. doi:10.1073/pnas.1112937108. 21885736 PMC3182706

[48] Biswal B, Yetkin FZ, Haughton VM, Hyde JS. Functional connectivity in the motor cortex of resting human brain using echo-planar MRI. Magn Reson Med 34: 537–541, 1995. doi:10.1002/mrm.1910340409. 8524021

[49] Braga RM, Buckner RL. Parallel interdigitated distributed networks within the individual estimated by intrinsic functional connectivity. Neuron 95: 457–471.e5, 2017. doi:10.1016/j.neuron.2017.06.038. 28728026 PMC5519493

[50] Gordon EM, Laumann TO, Gilmore AW, Newbold DJ, Greene DJ, Berg JJ, Ortega M, Hoyt-Drazen C, Gratton C, Sun H, Hampton JM, Coalson RS, Nguyen AL, McDermott KB, Shimony JS, Snyder AZ, Schlaggar BL, Petersen SE, Nelson SM, Dosenbach NU. Precision functional mapping of individual human brains. Neuron 95: 791–807.e7, 2017. doi:10.1016/j.neuron.2017.07.011. 28757305 PMC5576360

[51] Gordon EM, Chauvin RJ, Van AN, Rajesh A, Nielsen A, Newbold DJ, Lynch CJ, Seider NA, Krimmel SR, Scheidter KM, Monk J, Miller RL, Metoki A, Montez DF, Zheng A, Elbau I, Madison T, Nishino T, Myers MJ, Kaplan S, D'Andrea CB, Demeter DV, Feigelis M, Ramirez JSB, Xu T, Barch DM, Smyser CD, Rogers CE, Zimmermann J, Botteron KN, Pruett JR, Willie JT, Brunner P, Shimony JS, Kay BP, Marek S, Norris SA, Gratton C, Sylvester CM, Power JD, Liston C, Greene DJ, Roland JL, Petersen SE, Raichle ME, Laumann TO, Fair DA, Dosenbach NUF. A somato-cognitive action network alternates with effector regions in motor cortex. Nature 617: 351–359, 2023. doi:10.1038/s41586-023-05964-2. 37076628 PMC10172144

[52] Laumann TO, Gordon EM, Adeyemo B, Snyder AZ, Joo SJ, Chen M-Y, Gilmore AW, McDermott KB, Nelson SM, Dosenbach NU, Schlaggar BL, Mumford JA, Poldrack RA, Petersen SE. Functional system and areal organization of a highly sampled individual human brain. Neuron 87: 657–670, 2015. doi:10.1016/j.neuron.2015.06.037. 26212711 PMC4642864

[53] Noyce AL, Lefco RW, Brissenden JA, Tobyne SM, Shinn-Cunningham BG, Somers DC. Extended frontal networks for visual and auditory working memory. Cereb Cortex 32: 855–869, 2022. doi:10.1093/cercor/bhab249. 34467399 PMC8841551

[54] Reznik D, Trampel R, Weiskopf N, Witter MP, Doeller CF. Dissociating distinct cortical networks associated with subregions of the human medial temporal lobe using precision neuroimaging. Neuron 111: 2756–2772.e7, 2023. doi:10.1016/j.neuron.2023.05.029. 37390820

[55] Smith DM, Perez DC, Porter A, Dworetsky A, Gratton C. Light through the fog: using precision fMRI data to disentangle the neural substrates of cognitive control. Curr Opin Behav Sci 40: 19–26, 2021. doi:10.1016/j.cobeha.2020.12.004. 33553511 PMC7861476

[56] Nieto-Castañón A, Fedorenko E. Subject-specific functional localizers increase sensitivity and functional resolution of multi-subject analyses. NeuroImage 63: 1646–1669, 2012. doi:10.1016/j.neuroimage.2012.06.065. 22784644 PMC3477490

[57] DiNicola LM, Braga RM, Buckner RL. Parallel distributed networks dissociate episodic and social functions within the individual. J Neurophysiol 123: 1144–1179, 2020 [Erratum in J Neurophysiol 124: 307, 2020]. doi:10.1152/jn.00529.2019. 32049593 PMC7099479

[58] Buckner RL, DiNicola LM. The brain’s default network: updated anatomy, physiology and evolving insights. Nat Rev Neurosci 20: 593–608, 2019. doi:10.1038/s41583-019-0212-7. 31492945

[59] DiNicola LM, Buckner RL. Precision estimates of parallel distributed association networks: evidence for domain specialization and implications for evolution and development. Curr Opin Behav Sci 40: 120–129, 2021. doi:10.1016/j.cobeha.2021.03.029. 34263017 PMC8274557

[60] Saadon-Grosman N, Du J, Kosakowski HL, Angeli PA, DiNicola LM, Eldaief MC, Buckner RL. Within-individual organization of the human cognitive cerebellum: evidence for closely juxtaposed, functionally specialized regions (Preprint). *bioRxiv* 2023.12.18.572062, 2023. doi:10.1101/2023.12.18.572062. 38187706 PMC10769291

[61] Marek S, Siegel JS, Gordon EM, Raut RV, Gratton C, Newbold DJ, Ortega M, Laumann TO, Adeyemo B, Miller DB, Zheng A, Lopez KC, Berg JJ, Coalson RS, Nguyen AL, Dierker D, Van AN, Hoyt CR, McDermott KB, Norris SA, Shimony JS, Snyder AZ, Nelson SM, Barch DM, Schlaggar BL, Raichle ME, Petersen SE, Greene DJ, Dosenbach NU. Spatial and temporal organization of the individual human cerebellum. Neuron 100: 977–993.e7, 2018. doi:10.1016/j.neuron.2018.10.010. 30473014 PMC6351081

[62] Bostan AC, Strick PL. The basal ganglia and the cerebellum: nodes in an integrated network. Nat Rev Neurosci 19: 338–350, 2018. doi:10.1038/s41583-018-0002-7. 29643480 PMC6503669

[63] Goldman-Rakic PS. Topography of cognition: parallel distributed networks in primate association cortex. Annu Rev Neurosci 11: 137–156, 1988. doi:10.1146/annurev.ne.11.030188.001033. 3284439

[64] Mesulam MM. Large-scale neurocognitive networks and distributed processing for attention, language, and memory. Ann Neurol 28: 597–613, 1990. doi:10.1002/ana.410280502. 2260847

[65] Selemon LD, Goldman-Rakic PS. Common cortical and subcortical targets of the dorsolateral prefrontal and posterior parietal cortices in the rhesus monkey: evidence for a distributed neural network subserving spatially guided behavior. J Neurosci 8: 4049–4068, 1988. doi:10.1523/JNEUROSCI.08-11-04049.1988. 2846794 PMC6569486

[66] Buckner RL, Krienen FM, Yeo BT. Opportunities and limitations of intrinsic functional connectivity MRI. Nat Neurosci 16: 832–837, 2013. doi:10.1038/nn.3423. 23799476

[67] Fox MD, Raichle ME. Spontaneous fluctuations in brain activity observed with functional magnetic resonance imaging. Nat Rev Neurosci 8: 700–711, 2007. doi:10.1038/nrn2201. 17704812

[68] Murphy K, Birn RM, Bandettini PA. Resting-state fMRI confounds and cleanup. NeuroImage 80: 349–359, 2013. doi:10.1016/j.neuroimage.2013.04.001. 23571418 PMC3720818

[69] Power JD, Schlaggar BL, Petersen SE. Studying brain organization via spontaneous fMRI signal. Neuron 84: 681–696, 2014. doi:10.1016/j.neuron.2014.09.007. 25459408 PMC4254503

[70] Smith SM, Vidaurre D, Beckmann CF, Glasser MF, Jenkinson M, Miller KL, Nichols TE, Robinson EC, Salimi-Khorshidi G, Woolrich MW, Barch DM, Uğurbil K, Van Essen DC. Functional connectomics from resting-state fMRI. Trends Cogn Sci 17: 666–682, 2013. doi:10.1016/j.tics.2013.09.016. 24238796 PMC4004765

[71] Van Dijk KR, Hedden T, Venkataraman A, Evans KC, Lazar SW, Buckner RL. Intrinsic functional connectivity as a tool for human connectomics: theory, properties, and optimization. J Neurophysiol 103: 297–321, 2010. doi:10.1152/jn.00783.2009. 19889849 PMC2807224

[72] Fedorenko E, Duncan J, Kanwisher N. Broad domain generality in focal regions of frontal and parietal cortex. Proc Natl Acad Sci USA 110: 16616–16621, 2013. doi:10.1073/pnas.1315235110. 24062451 PMC3799302

[73] Duncan J. The multiple-demand (MD) system of the primate brain: mental programs for intelligent behaviour. Trends Cogn Sci 14: 172–179, 2010. doi:10.1016/j.tics.2010.01.004. 20171926

[74] Jacoby N, Bruneau E, Koster-Hale J, Saxe R. Localizing pain matrix and theory of mind networks with both verbal and non-verbal stimuli. NeuroImage 126: 39–48, 2016. doi:10.1016/j.neuroimage.2015.11.025. 26589334 PMC4733571

[75] Cui DM, Yan YJ, Lynch JC. Pursuit subregion of the frontal eye field projects to the caudate nucleus in monkeys. J Neurophysiol 89: 2678–2684, 2003. doi:10.1152/jn.00501.2002. 12612013

[76] Leichnetz GR. Connections of the medial posterior parietal cortex (area 7m) in the monkey. Anat Rec 263: 215–236, 2001. doi:10.1002/ar.1082. 11360237

[77] Parthasarathy HB, Schall JD, Graybiel AM. Distributed but convergent ordering of corticostriatal projections: analysis of the frontal eye field and the supplementary eye field in the macaque monkey. J Neurosci 12: 4468–4488, 1992. doi:10.1523/JNEUROSCI.12-11-04468.1992. 1279139 PMC6575998

[78] Shook BL, Schlag-Rey M, Schlag J. Primate supplementary eye field. II. Comparative aspects of connections with the thalamus, corpus striatum, and related forebrain nuclei. J Comp Neurol 307: 562–583, 1991. doi:10.1002/cne.903070405. 1869632

[79] Haber SN, Fudge JL, McFarland NR. Striatonigrostriatal pathways in primates form an ascending spiral from the shell to the dorsolateral striatum. J Neurosci 20: 2369–2382, 2000. doi:10.1523/JNEUROSCI.20-06-02369.2000. 10704511 PMC6772499

[80] Haber SN, Knutson B. The reward circuit: linking primate anatomy and human imaging. Neuropsychopharmacology 35: 4–26, 2010. doi:10.1038/npp.2009.129. 19812543 PMC3055449

[81] Heimer L. The legacy of the silver methods and the new anatomy of the basal forebrain: implications for neuropsychiatry and drug abuse. Scand J Psychol 44: 189–201, 2003. doi:10.1111/1467-9450.00336. 12914582

